# Environmental (e)RNA advances the reliability of eDNA by predicting its age

**DOI:** 10.1038/s41598-021-82205-4

**Published:** 2021-02-02

**Authors:** Nathaniel T. Marshall, Henry A. Vanderploeg, Subba Rao Chaganti

**Affiliations:** 1 Cooperative Institute for Great Lakes Research, School for Environment and Sustainability, University of Michigan, 440 Church Street, Ann Arbor, MI 48109 USA; 2 National Oceanic and Atmospheric Administration, Great Lakes Environmental Research Laboratory, 4840 S. State Rd, Ann Arbor, MI 48108 USA

**Keywords:** Genetic models, Invasive species, Ecological genetics, Molecular ecology, Genetic markers, Conservation genomics, Invasive species, Environmental impact

## Abstract

Environmental DNA (eDNA) analysis has advanced conservation biology and biodiversity management. However, accurate estimation of age and origin of eDNA is complicated by particle transport and the presence of legacy genetic material, which can obscure accurate interpretation of eDNA detection and quantification. To understand the state of genomic material within the environment, we investigated the degradation relationships between (a) size of fragments (long vs short), (b) genomic origins (mitochondrial vs nuclear), (c) nucleic acids (eDNA vs eRNA), and (d) RNA types (messenger (m)RNA vs ribosomal (r)RNA) from non-indigenous *Dreissena* mussels. Initial concentrations of eRNA followed expected transcriptional trends, with rRNAs found at > 1000 × that of eDNA, and a mitosis-associated mRNA falling below detection limits within 24 h. Furthermore, the ratio of eRNA:eDNA significantly decreased throughout degradation, potentially providing an estimate for the age of genomic material. Thus, eRNA quantification can increase detection due to the high concentrations of rRNAs. Furthermore, it may improve interpretation of positive detections through the eRNA:eDNA ratio and/or by detecting low abundant mitosis-associated mRNAs that degrade within ~ 24 h.

## Introduction

Environmental (e)DNA (i.e., genetic material from urine, waste, mucus, or sloughed cells) collected from aquatic habitats has improved the management and assessment of a species’ distribution and entire community compositions^1,2^. The analysis of eDNA has quickly become a powerful tool for improving species detection and unraveling patterns of biodiversity^3–5^. Standardized methods are now being employed for the development and implementation of quantitative (q)PCR that can measure the amount of target genetic material within a sample^6–8^. However, relating the quantification of eDNA to its original source is complicated due to the complex interactions of the temporal, physical, and chemical factors that influence the degradation of eDNA within the environment^9,10^.

Environmental DNA can be transported within the water column in both lotic and lentic systems^11–13^, which may result in improper interpretation of a species’ spatiotemporal distribution. Furthermore, a number of mesocosm studies have detected eDNA for many months^14–16^, signifying the potential for false positive detections resulting from “legacy” eDNA. Degradation of eDNA mainly depends on abiotic factors, such as water temperature, pH, salinity, and ultraviolet (UV) radiation, and biotic factors, such as microbes and activity of extracellular enzymes^9^. Additionally, degradation of eDNA is likely to be tied to the genomic characteristics of the target region, such as length of the fragment, genomic origin (nuclear or mitochondrial), and the nucleic acid composition (RNA or DNA)^9,17–19^. However, studies comparing environmental degradation rates across the genomic origins are scarce, and knowledge about gene-dependent detection and quantification is necessary for advancing eDNA methodologies.

Genetic material is predicted to shear at random, resulting in long fragments degrading faster than short fragments^20^, and thus eDNA has largely been restricted to short markers of < 250 base pairs (bp)^2,21^. Additionally, the genetic state of eDNA in the natural environment is composed of both nuclear (nu-DNA) and mitochondrial (mt-DNA) genomes, which differ in their structure, and potentially their abundance and detectability^22,23^. Majority of species-specific eDNA markers use mt-DNA (typically targeting the cytochrome oxidase subunit I (COI) or cytochrome b (Cyt*b*) genes), due to large existing sequence databases^2^, expected higher density of mt-DNA compared to nu-DNA (10–1000 s of mitochondria to a single nucleus per cell)^20^, and the assumption that the mt-genome is more stable due to it’s circular structure^24^. However, repetitive ribosomal nu-DNA genes, such as the small (18S) and large subunits (28S), can occur at similar or even higher copies than mt-DNA in living and sloughed cellular material^22,25^, suggesting nu-DNA may increase eDNA detection rates for some taxa.

Furthermore, due to its conformation as a single-stranded structure and the presence of hydroxyl groups increasing abiotic chemical break down^26–28^, RNA has long been considered less stable than its DNA counterpart. Thus, the predicted quick breakdown of eRNA may reduce false positives related to eDNA transport and legacy signal^17^, as RNA degrades rapidly after cell death^29,30^. Within eukaryotic cells, ribosomal (r)RNAs comprise > 80% of the total RNAs within a cell^31,32^, and thus are predicted to be found in greater concentrations than that of messenger (m)RNA. Additionally, rRNA is hypothesized to be less susceptible to degradation compared to mRNA due to structure stability^33^, and thus gene detection within eRNA is likely dependent upon the RNA type. Therefore, an accurate interpretation of environmental genetic signal is dependent upon knowledge of the shedding and degradation rates between the nu- and mt-genomes, between eDNA and eRNA, and between the RNA types within eRNA.

**Figure 1:**
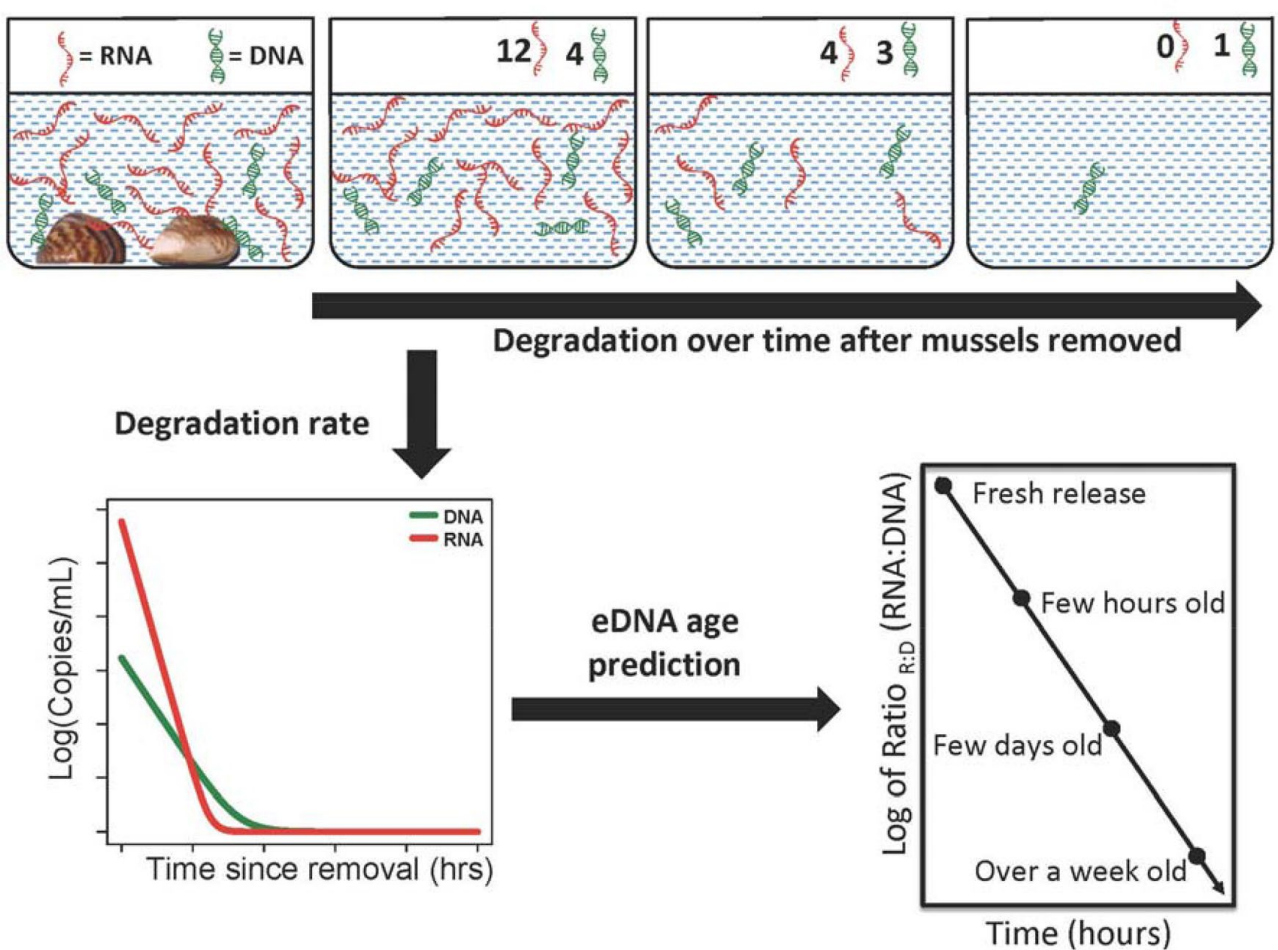
Schematic of experimental design, in which the degradation rates of environmental (e)DNA and (e)RNA were compared across multiple genes collected in environmental genomic material released from dreissenid mussels. The concentration of eRNA depletes faster than eDNA, providing a predictor for estimating time since genomic material release.

Knowledge of the state of genetic material in natural environments, including distribution of genomes and the predominantly available genes, is important for proper marker design that can accurately detect and distinguish fresh from legacy eDNA, and potentially decrease noise from eDNA transport. We hypothesize degradation rates will vary across genomic origin, with long fragments degrading faster than short fragments, nu-DNA degrading faster than mt-DNA, eRNA degrading faster than eDNA, and mRNA degrading faster than rRNA. Further we hypothesize based on the DNA and RNA degradation ratios we can predict the age of eDNA and avoid potential false positives (Fig. 1). Hence the current study investigates the degradation relationships between (a) size of fragments (long vs short markers), (b) genomic origins (mt-DNA vs nu-DNA), (c) nucleic acids (eDNA vs eRNA), and (d) RNA types (mRNA vs rRNA) across varying densities of the non-indigenous dreissenid mussels (zebra (*Dreissena polymorpha*) and quagga mussels (*D. rostriformis bugensis*)) for which early detection and estimates of biomass related impacts are important^34^. The results of this study will advance eDNA methodology and improve the reliability of environmental genetic sampling, by understanding rates of release and degradation between nucleic acids across the mitochondrial and nuclear genomes.

## Results

For both eDNA and eRNA, four gene regions were selected across the mt- (16S and COI) and nu-genomes (18S and histone producing H2B) (Table 1). These gene regions target both mRNA (COI and H2B) and rRNA (16S and 18S) (Table 1). Furthermore, for eDNA, two of these gene regions (16S and H2B) were analyzed for fragments of varying length (Table 1). For all the target markers, qPCR efficiencies ranged from 94.3 to 103.2 (Table 1). There was no amplification in any of the laboratory controls, filtration controls, or PCR no-DNA template controls during the experiment.

**Table 1:** Dreissenid specific primer pairs (F1-Forward 1, F2-Forward 2, and R-Reverse) used for quantitative PCR analysis of environmental DNA and RNA degradation.

Gene	Genome	Primer sequence	Source	Tm	Length	Efficiency	R^2^
16S	mt rRNA	**F1:** GTTAATAGCTGTGCTAAGGTAGC	Current Study	63 °C	341	94.4	0.994
		**F2:** TGGGGCAGTAAGAAGAAAAAAATAA	^47^	62 °C	141	95.3	0.995
		**R:** CATCGAGGTCGCAAACCG	^47^	67 °C	–	–	–
COI	mt mRNA	**F:** ATTTTATCTCTTCATATYGGGGGAGC	Current study	64–66 °C	128	94.3	0.998
		**R:** CCAATAGAWGTRCARAACAAAGG	Current study	59–64 °C	–	–	–
18S^a^	nu rRNA	**F:** AACYCGTGGTGACTCTGGAC	^58^	67–70 °C	169	94.3	0.995
		**R:** GTGTCTCATGCTCCCTCTCC	^58^	67 °C	–	–	–
H2B	nu mRNA	**F1:** CGCGCGCTCCACTGACAAGA	^46^	73 °C	251	98.2	0.989
		**F2:** TTGCCCACTACAACAAGCGA	Current study	67 °C	75	103.2	0.997
		**R:** CACCAGGCAGCAGGAGACGC	^46^	74 °C	–	–	–

The genomic origin (mitochondrial (mt) or nuclear (nu)) and RNA types (ribosomal (r)RNA or messenger (m)RNA) are listed for each gene, as well as the length (base pairs), melting temperature (Tm), primer efficiency (%), and R^2^ for each primer set.

^a^Adopted from a primer set designed from loop-mediated isothermal amplification.

### Initial concentration at time 0

**Figure 2:**
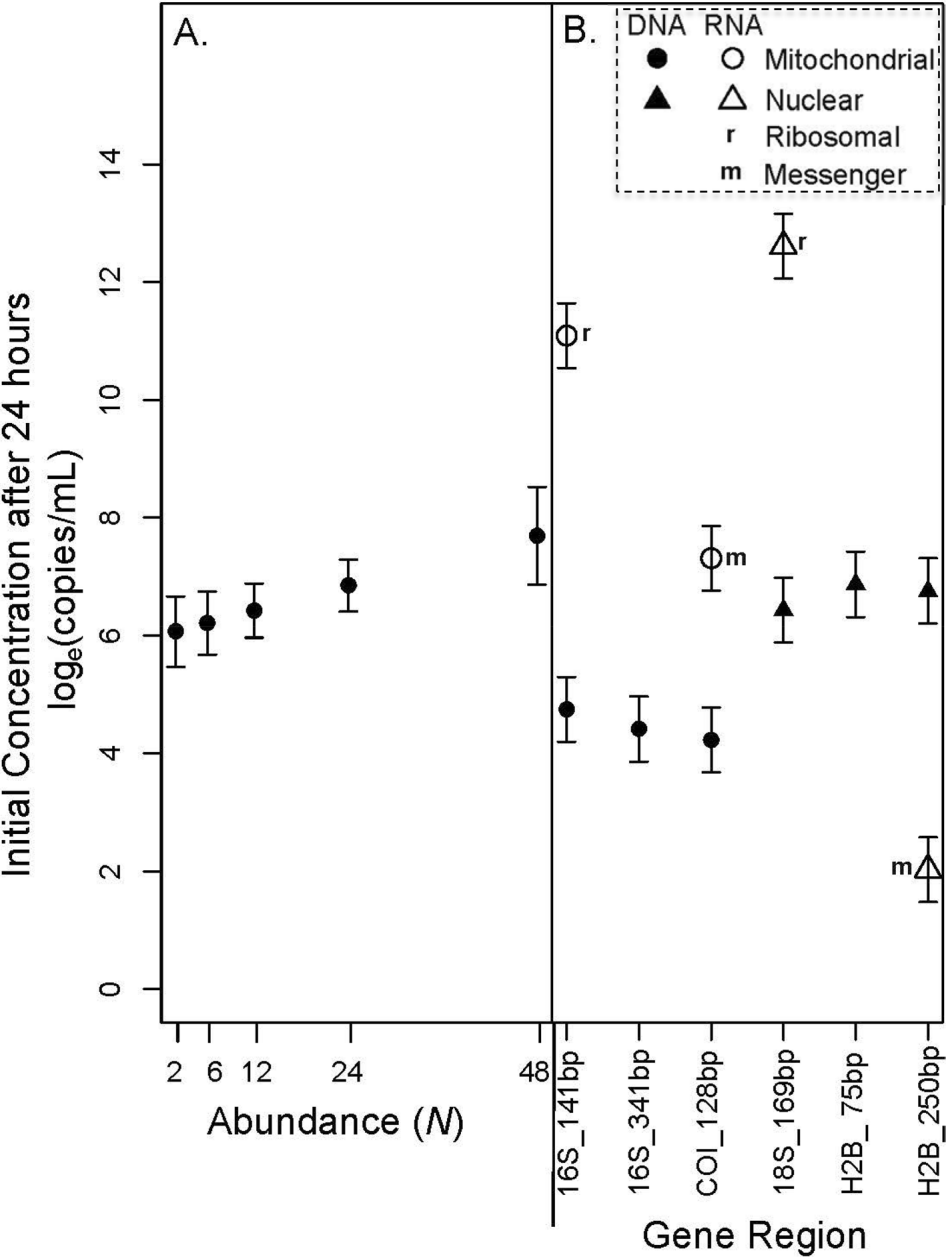
Shedding concentration log_e_(copies/mL) released after 24 h analyzed across (**A**) mussel abundance with all genetic markers combined, and (**B**) across each of the target gene markers with all abundances combined. Interactions between gene marker and abundance are in the Supplementary Information, including Supplementary Fig. S1. Error bars represent 95% confidence intervals.

The concentration at time 0 increased with mussel abundance across all genes for both eDNA and eRNA (Fig. 2A, Supplementary Figs. S1, S2; Supplementary Tables S2–S5; *p* = 0.009**; Supplementary Table S3). There was no difference in initial concentrations between size of fragments of eDNA for either mt-16S or nu-H2B (Fig. 2B; Table 2, Supplementary Tables S5–S8). The initial concentration slightly differed across genomic origins, with nu-DNA released in higher concentration than mt-DNA (p < 0.001***; Fig. 2B; Table 2, Supplementary Tables S6, S8).

**Table 2:** Initial concentration released over 24 h, linear mixed-effect model decay constants (± SE), half-life range estimated from 95% confidence interval, and number (*N*) of detections at 144 and 240 h for each gene target across eDNA and eRNA.

Gene target	Initial concentration (log_e_ (copies/mL/24 h))	Decay constant (per hour)	Half-life (h)	*N* detections (out of 13)	
				144 h	240 h
mt16S_141bp DNA	4.75 ± 0.272	− 0.0378 ± 0.0025	16.23–21.07	9 (69%)	2 (15%)
mt16S_341bp DNA	4.42 ± 0.272	− 0.0420 ± 0.0025	14.78–18.68	8 (62%)	3 (23%)
mtCOI_128bp DNA	4.23 ± 0.272	− 0.0398 ± 0.0025	15.47–19.86	1 (8%)	0 (0%)
nu18S_169bp DNA	6.43 ± 0.272	− 0.0466 ± 0.0027	13.38–16.78	8 (62%)	2 (15%)
nuH2B_75bp DNA	6.87 ± 0.272	− 0.0461 ± 0.0025	13.59–16.78	11 (85%)	4 (31%)
nuH2B_250bp DNA	6.75 ± 0.272	− 0.0480 ± 0.0027	13.00–16.02	8 (62%)	2 (15%)
mt16S_141bp rRNA	11.09 ± 0.272	− 0.0561 ± 0.0025	11.36–13.54	12 (92%)	10 (69%)
mtCOI_128bp mRNA	7.31 ± 0.272	− 0.0602 ± 0.0025	10.65–12.51	0 (0%)	0 (0%)
nu18S_169bp rRNA	12.61 ± 0.272	− 0.0735 ± 0.0025	8.84–10.09	13 (100%)	13 (100%)
nuH2B_250bp mRNA	2.03 ± 0.272	–	–	0/0 (0/0)	0 (0%)

Considerably higher concentrations were found for eRNA molecules compared to eDNA molecules for the mt-16S (> 1000 × more eRNA; *p* < 0.001***), mt-COI (~ 30 × more eRNA; *p* < 0.001***), and nu-18S genes (> 1000 × more eRNA; *p* < 0.001***; Fig. 2B; Table 2, Supplementary Tables S6, S8). The nu-H2B was the only gene to display substantially less eRNA compared to eDNA (~ 1000 × more eDNA; *p* < 0.001***; Fig. 2B; Table 2, Supplementary Tables S4, S5). Ribosomal RNA genes (mt-16S and nu-18S) had greater concentrations than messenger genes (mt-COI and nu-H2B) at time 0 (*p* < 0.001***; Fig. 2B; Table 2, Supplementary Tables S6, S8).

### Degradation

Five of the six eDNA targets were detected throughout the entirety of the experiment (i.e., 240 h), with the COI region as the only exception (Table 2). However, detection for each marker occurred in ≤ 4 tanks (15.38–30.77%) at 240 h compared to 8–11 tanks (61.54–84.62%) at 144 h (Table 2). For eRNA, only the rRNA gene targets (16S and 18S) were detected past 72 h, (92.31% and 100% detection at 144 h; 69.23% and 100% detection at 240 h; Table 2). The COI mRNA gene target went undetected after 72 h, while the H2B mRNA gene was often undetected after 24 h, with only a 23.08% detection at 48 h.

**Figure 3:**
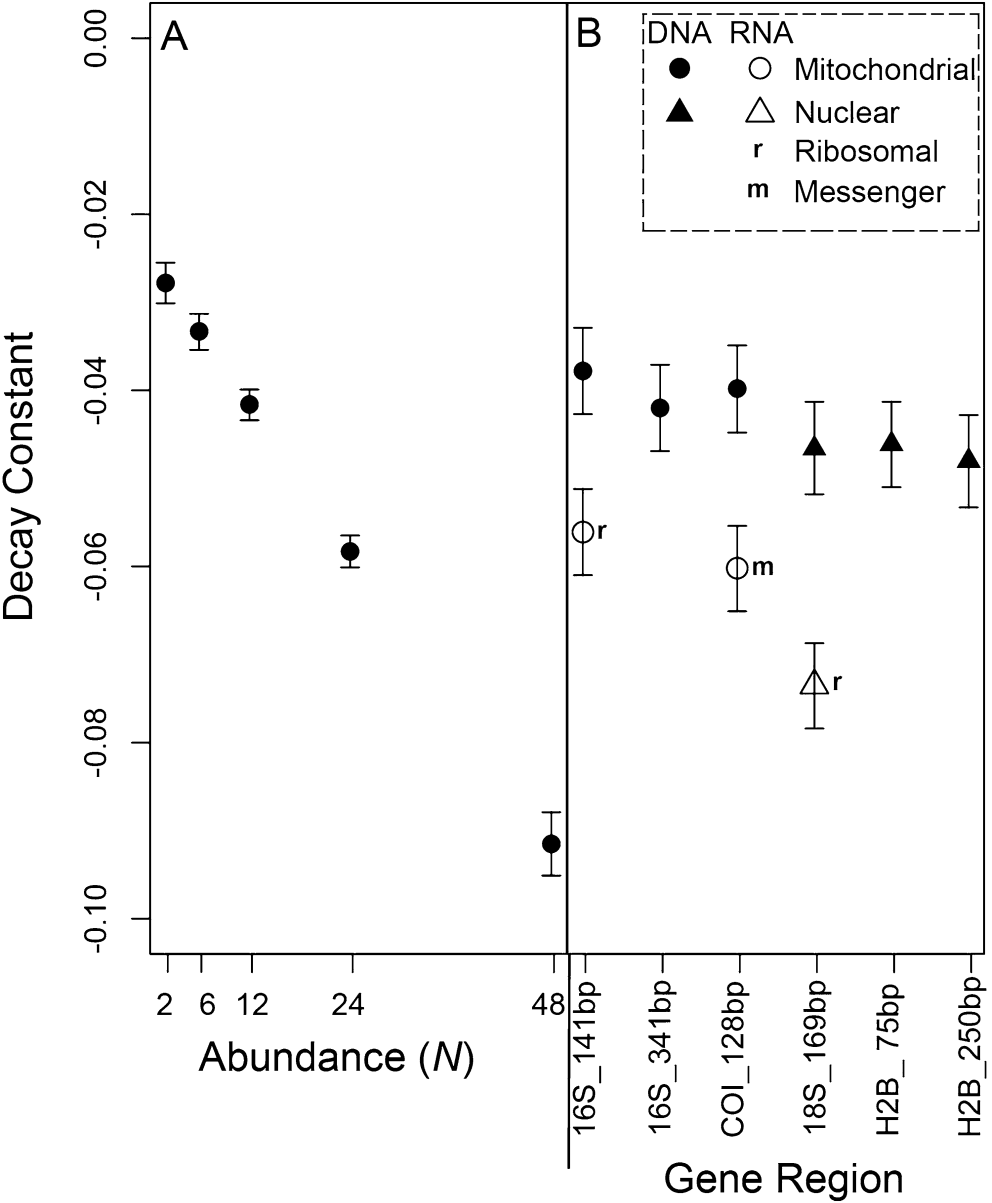
Decay constant calculated from a first-order exponential decay model across times 0 to 72 h for (**A**) mussel abundance with all genetic markers combined, and (**B**) across each of the target gene markers with all abundances combined. Interactions between gene marker and abundance are in the Supplementary Information, including Supplementary Fig. S3. Error bars represent 95% confidence intervals.

**Figure 4:**
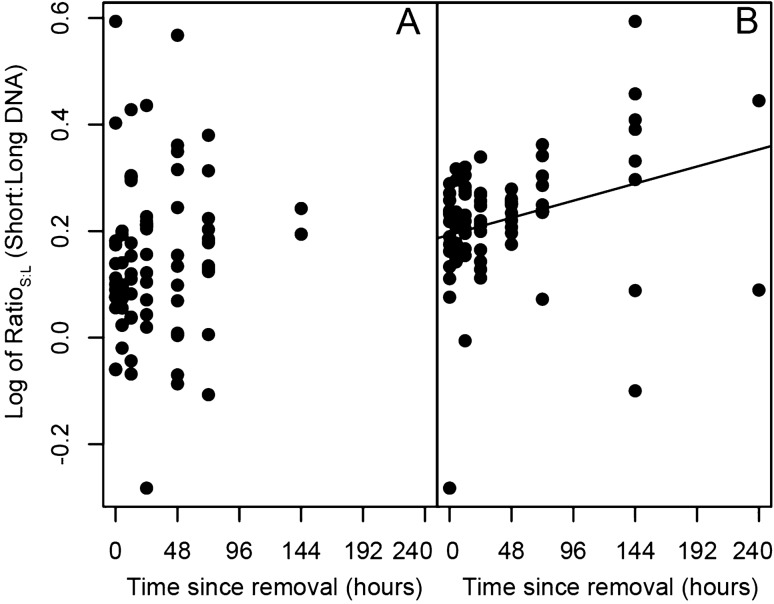
Temporal changes in log_10_ Ratio_S:L_ (short:long) for (**A**) 16S mitochondrial (mt) ribosomal (r)DNA (*p* > 0.05) and (**B**) H2B nuclear (nu) messenger (m)DNA (*p* = 0.004**, R^2^ = 0.09).

Overall, decay constants significantly increased with mussel abundance across all genes for both eDNA and eRNA (*p* < 0.001***; Fig. 3A; Supplementary Tables S9–S11). Decay constants did not differ across size of fragments within either the mitochondrial or nuclear genes for eDNA (Fig. 3B, Supplementary Figs. S3, S4; Table 2, Supplementary Tables S12–S16), however, there was a small increase in the ratio of short:long eDNA fragments (Ratio_S:L_) over the course of degradation for the H2B gene (Fig. 4). Across eDNA, the nu-DNA and mt-DNA genes had large overlap in decay constants, with nu-DNA displaying a slightly faster half-life (Fig. 3B, Supplementary Figs. S3, S4; Table 2, Supplementary Tables S13, S15).

**Figure 5:**
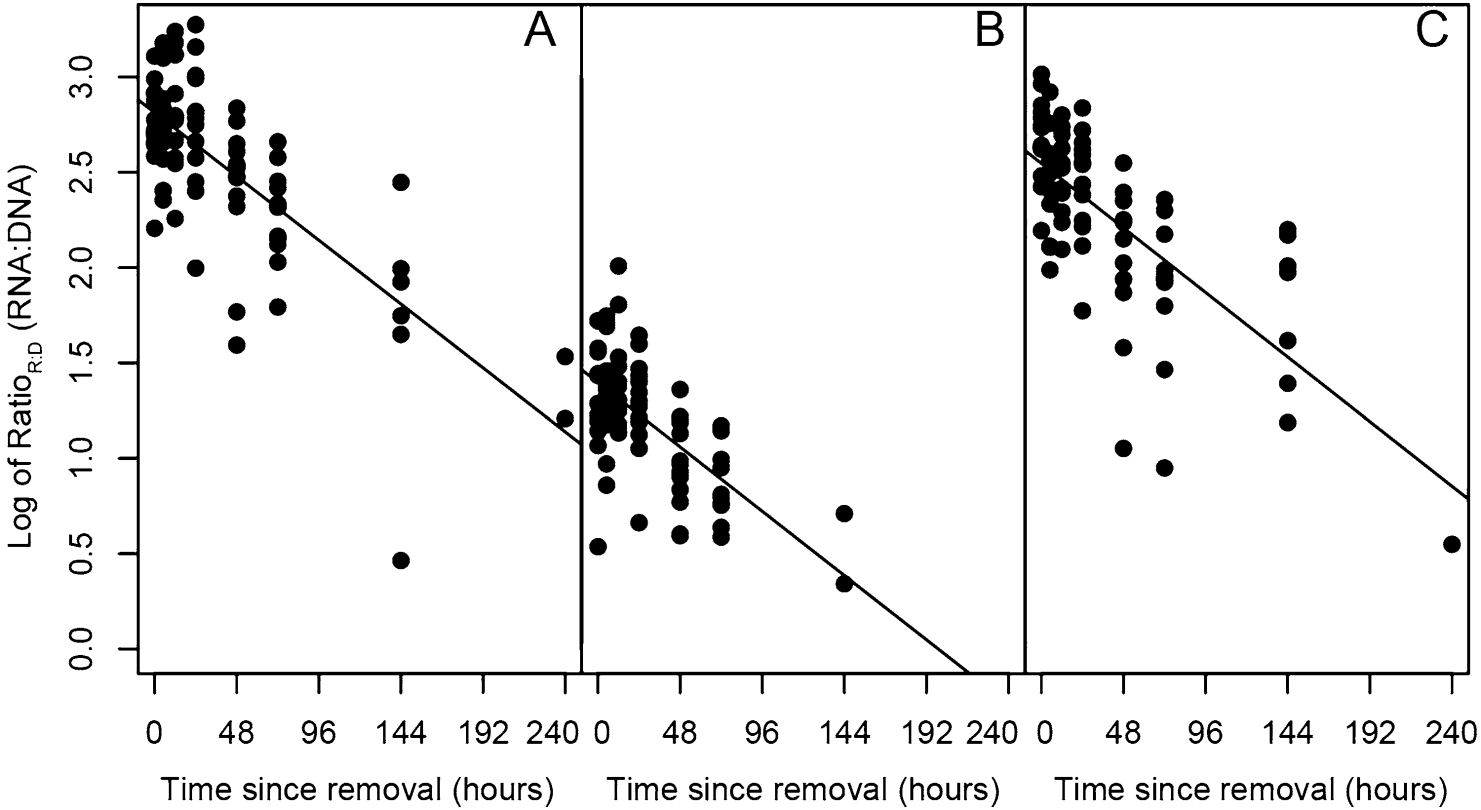
Temporal changes in log_10_ Ratio_R:D_ (RNA:DNA) for (**A**) 16S mitochondrial (mt) ribosomal (r)RNA (*p* < 0.001***, R^2^ = 0.54), (**B**) COI mitochondrial (mt) messenger (m)RNA (*p* < 0.001***, R^2^ = 0.40), and (**C**) 18S nuclear (nu) rRNA (*p* < 0.001***, R^2^ = 0.49).

Due to the low concentrations of the H2B gene found within eRNA, the decay constant was not calculated for H2B eRNA. The other three RNA markers displayed faster decay constants than their DNA counterparts (all *p* < 0.001***; Fig. 3B, Supplementary Figs. S3, S4; Table 2, Supplementary Tables S13, S15). Furthermore, the ratio of eRNA:eDNA (Ratio_R:D_) substantially decreased for all genes over the course of degradation (p < 0.001***; Fig. 5). Among eRNA, the mitochondrial genes (16S and COI) displayed similar decay constants, while the nuclear gene (18S) displayed a significantly faster decay compared to mitochondrial genes (*p*  <  0.005**; Fig. 3B; Table 2).

## Discussion

We present one of the first investigations into unraveling the release and degradation dynamics from environmental genomic material through a multi-gene approach targeting both eDNA and eRNA across the mitochondrial and nuclear genomes. Our findings present an informative characterization of the relationships between genomic fragments found within environmental samples, and furthermore, how these relationships change over the course of degradation. Environmental DNA shedding and degradation rates are known to display a positive association with organism abundance or biomass^35–40^, and this is the first study to display similar trends for eRNA across mt- and nu-genes.

Degradation rates of long and short size fragments have varied across studies, where Jo et al.^40^ demonstrated faster degradation of longer fragments (719 bp vs 127 bp of the Cyt*b* region), while Bylemans et al.^37^ determined similar decay rates across three size fragments (96, 285, and 515 bps of the COI region). In the present study, there was no difference in shedding or decay constants related to fragment length for either a mitochondrial (16S: 141 and 341 bp) or nuclear gene (H2B: 75 and 251 bp). Although, across the course of the experiment, the Ratio_S:L_ for the H2B gene increased form ~ 1.5 to ~ 2.5, suggesting a slightly faster degradation of the long fragment. However, this displayed a very weak relationship (R^2^ = 0.09) and a very minute increase over the 240 h experiment, which is unlikely to improve age estimates from environmental samples. A stronger relationship might be found with longer fragments (e.g., > 700 bps), which are more susceptible to degradation effects. However qPCR greatly reduces in amplification efficiency with target regions > 400 bp, thereby reducing accuracy in quantification and leading to biased estimates for longer size fragments. For example, Bylemans et al.^37^ displayed a qPCR efficiency of 72.2% for a 515 bp fragment, well outside the recommend range of 90–110%^41^. Previous size fractionation water sampling determined that eDNA transitions from an intracellular to a subcellular state over the course of degradation^35^. Therefore, the degradation of eDNA into smaller size fragments might not occur until the subcellular stage. In the present study, eDNA material was captured on a 1.2 µm filter, and thus the genetic signal from the subcellular state might have been lost. Size fractionation with a smaller pore size may provide a true signal of size dependent degradation through the capture of this subcellular material.

It has widely been considered that mt-eDNA is more abundant than nu-eDNA fragments, due to higher copy numbers per cell (2–10 mt-genomes/mitochondria and 10–1000 s of mitochondria/cell)^42^ and the mt-genome circular plasmid exhibiting less susceptibility to decay^20,24^. However, this trend has yielded contrary results within eDNA mesocosm studies. For example, Bylemans et al.^37^ determined that mt-eDNA (COI) was shed in greater concentration than nu-eDNA (ITS) from mesocosms of Goldfish (*Carassius auratus*), while Moushomi et al.^43^ found that nu-eDNA (18S) was more abundant than mt-eDNA (COI) from mesocosms of *Daphnia magna*, and Jo et al.^44^ found similar concentrations between nu-eDNA (ITS) and mt-eDNA (Cyt*b*) from mesocosms of Pacific Jack Mackerel (*Trachurus symmetricus*). Distinct differences between releases of mt- and nu-genomic material across various studies might be a result of physiological differences between study organisms, age of organisms, or even stress of organisms. For example, Jo et al.^44^ determined nu-eDNA was released faster than mt-eDNA for large-older fish. Similar to Moushomi et al.^43^, we found the multicopy nu-eDNA (18S and H2B) in higher concentrations than that of mt-eDNA (16S and COI), and found these relationships did not change across mussel abundances. Furthermore, work with five species of marine bivalves within the genus *Mytilus,* calculated an average of 212 copies per cell for the H2B gene^45^, supporting the high concentrations of the H2B gene found within eDNA in our study. However it is important to note that we did not collect data to calculate true shedding rates, but rather just the concentrations released after 24 h. Regardless of copy number per cell, both mt- and nu-genes have successfully detected dreissenid mussels from environmental DNA in rivers and lakes^46–48^, suggesting adequate release of both genomes for eDNA detection.

RNA has been considered a less stable molecule than DNA, due to its conformation as a single-stranded structure and the presence of hydroxyl groups increasing abiotic chemical break down^26–28^. Thus, eRNA has been proposed as a more time-sensitive environmental marker than eDNA^17^. The only previous study to analyze release and degradation rates of eRNA, found significantly lower release concentrations of eRNA compared to eDNA for a COI marker targeting two marine fan worms^49^. Contrary, here the initial concentrations of eRNA were greater than eDNA for three of the four genes investigated, including the previously analyzed COI region (in addition to the 16S rRNA and 18S rRNA). Methodological differences might have been a key factor in altering these results, as this study differed in extraction protocol and the cDNA synthesis enzyme. These changes to the protocol likely result in differences in eRNA extraction and cDNA synthesis efficiencies, which could alter the quantification of eRNA between studies. Furthermore, as discussed earlier, physiological differences between species, and their levels of stress, may result in varying concentrations of genetic material, including different gene targets. Future studies should aim to use common protocols across various taxa, to examine similarities and differences in eRNA detection and degradation across species.

The histone producing mRNA H2B gene was the single gene region to display lower concentrations of eRNA compared to eDNA, which was often undetected after 24 h within eRNA but could be detected at 10 days with eDNA. The H2B gene is highly expressed during the S-phase of mitosis^50^, and thus is predicted to be in low RNA concentrations within sloughed dead/dying cellular material, which would not be actively performing mitosis. This is the first study to investigate eRNA concentrations across ribosomal and messenger RNAs, in which the rRNA genes (mt-RNA 16S and nu-RNA 18S) displayed significantly greater concentrations. This pattern corresponds to transcriptional relationships within eukaryotic cells, as rRNA comprises > 80% of the total RNAs within a cell^31,32^. Additionally, these trends were upheld in all abundance treatments, suggesting no abundance or biomass related impacts. Considering these two rRNA genes had significantly higher concentrations, and were the highest detected molecules at the 10-day sampling time point, future eDNA programs can improve detection of low abundant organisms, by targeting these highly concentrated rRNAs.

Contrary to Wood et al.^49^, we found measurable differences in the decay rates between eDNA and eRNA for the three investigated gene regions. All three RNA molecules displayed an ~ 4–5 h faster half-life than their DNA counterparts, with this pattern upheld across abundances. Furthermore, the Ratio_R:D_ for all three gene regions decreased by an order of magnitudes over the course of degradation. Both rRNA markers decreased from > 1000 × to ~ 10 × that of DNA, likewise the mRNA decreased from > 30 × to ~ 1 × that of DNA, suggesting significantly greater degradation of RNA compared to DNA across both mitochondrial and nuclear genes. Therefore, the Ratio_R:D_ has the potential to be a predictor of the age of genomic material, thereby greatly improving interpretation of detections related to legacy or transported genomic material. However, it is important to note that within the natural environment, multiple individuals and populations will contribute to the entire pool of genomic material over a spatiotemporal scale. Therefore, our mesocosm study may oversimplify the complex temporal mixing of old and new eDNA/eRNA, which will complicate the Ratio_R:D._ On the other hand, the two mRNA gene targets were the quickest molecules to reach levels of non-detection, with the mitosis-associated mRNA often undetected within 24 h. Thus, a positive detection of this marker would be a strong indicator of a recent (within ~ 24 h) living organism within the sampled area.

Ribosomal RNA is hypothesized to be less susceptible to degradation compared to mRNA due to structure stability^33^, however here the decay constants were similar between the mitochondrial rRNA (16S) and mRNA (COI) molecules. Similar to the decay comparisons between genomic origins of eDNA, the nuclear rRNA 18S gene displayed a faster decay than either of the mitochondrial genes (16S and COI). This faster degradation of nuclear eDNA and eRNA compared to mitochondrial eDNA and eRNA, suggests overall faster degradation of nuclear genomic material, possibly due to the increased stability of the mitochondria. Although, both nuclear and mitochondrial eDNA and eRNA were detected throughout the span of the experiment, suggesting other factors maybe important for length of detectability.

In summary, these results demonstrate how incorporating eRNA quantification into existing eDNA protocols can both increase detection probability by targeting highly concentrated rRNAs and improve interpretation of positive detections by modeling the ratio of eRNA:eDNA, or assessing detection of low abundant mitosis-associated mRNAs. The release and degradation relationships between genomic fragments found here might change due to physiological differences between life-histories^51^ or between organisms^19^, and thus these genomic relationships should be investigated for other taxonomic groups. We found a significant decrease in the Ratio_R:D_ over time, allowing us to predict the age of genomic material, and thus reduce error from legacy genomic signal. By understating how to interpret detection and quantification of markers from varying genomic origins, we undoubtedly enhance environmental genomic monitoring across spatial and temporal scales.

## Methods

### Experimental design

Quagga and zebra mussels were scraped by hand from National Oceanic and Atmospheric Administration (NOAA) monitoring buoys in western Lake Erie (October 18, 2019), and immediately returned to NOAA Great Lakes Environmental Research Laboratory in wrapped lake water-soaked paper-towel in coolers. In the laboratory, dreissenid shells were scraped and rinsed with diH_2_O to remove any sediment material from their surface. Approximately 300 cleaned dreissenid mussels were acclimated to the lab in two 20 L aquaria filled with mussel media hard water (diH_2_O containing 1.5 mM NaHCO_3_, 0.5 mM MgSO_4_, and 0.75 mM CaCl_2_) that has been used for long-term maintenance of mussels in the laboratory. The aquaria were maintained at room temperature (~ 20 °C) with continuous aeration for two days. The mussels were feed frozen algae (freshwater *Nannochloropsis* (https://reedmariculture.com/product_instant_algae_nanno_3600.php)) twice in the first 48 h, before being moved into the experimental mesocosms.

Sixteen 18.9 L mesocosm tanks were thoroughly cleaned with detergent and bleached with a 0.1% sodium hypochlorite solution, followed by multiple rinses with diH_2_O. The mesocosms were filled with 15 L of mussel media hard water, and maintained at room temperature (~ 20 °C) with continuous aeration. Five abundance treatments of three replicates (total 15 mesocosms) were designed of varying dreissenid abundance. These abundance treatments consisted of 2, 6, 12, 24, and 48 mussels, with each mesocosm consisting of equal numbers of zebra and quagga mussels. The sixteenth mesocosm contained no mussels, to act as a laboratory negative control. Each tank was covered with saran wrap for the duration of the experiment. The organisms were maintained within the tanks without feeding for 24 h, after which they were removed by gloved hand. A new glove was worn for each tank.

### Water collection and processing

A control water sample was collected from each tank prior to the addition of organisms, to test for pre-experiment contamination. The time after removing the mussels from each tank was defined as time 0, and water samples were collected from each tank thereafter, at hour 0, 5, 12, 24, 48, 72, 144 (6 days), and 240 (10 days). Water samples (100 mL) were collected ~ 4 cm below the surface by filling a 50 mL falcon tube twice, after mixing the water by hand for ~ 3–5 s. A new glove was worn for each tank. Environmental DNA and eRNA was sampled using a 47-mm-diameter glass microfiber filter GF/C (nominal pore size 1.2 µm; GE Healthcare Life Science, MA, USA). The filtering devices (i.e., filter cups, plastic holders, 1 L beakers, and tweezers) were bleached after every use in 0.1% sodium hypochlorite solution for at least 5 min. After water filtration, the GF/C filters were split into two equal halves and immediately placed into an – 80 °C freezer until DNA/RNA extraction. At each sampling time two negative controls were collected, a 50 mL diH_2_O as a filtration negative control, and 100 mL from the negative control mesocosm.

### eDNA and eRNA extraction and cDNA synthesis

The eDNA and eRNA were simultaneously extracted from one of the two filter half’s using a *Quick*-DNA/RNA Miniprep Plus Kit (Zymo Research, CA, USA), with a modified extraction protocol. Briefly, 700 µL of 1X RNA Shield, 70 µL of PK Digestion Buffer, and 35 µL of Proteinase K were added to completely submerse each half filter in a 2 mL screw-cap tube, and incubated at 55 °C for 1 h. After incubation, the liquid and filter were placed into a QiaShredder spin column (Qiagen, MD, USA), and the flow through was processed continuing with the *Quick*-DNA/RNA Miniprep Plus Kit following the manufacturer's protocol, including a DNase treatment (DNase I enzyme, Zymo Research) for the eRNA extractions. The total eDNA or eRNA was eluted in 75 µL DNase/RNase-free water. All eDNA extractions were placed in a freezer (− 20 °C) until qPCR analysis, while eRNA extractions were immediately prepared for cDNA synthesis. A negative extraction control was included with each set of extractions.

eRNA extractions were transcribed into cDNA using SuperScript IV VILO Master Mix (ThermoFisher Scientific, MA, USA). The SuperScript IV VILO Master Mix includes oligo (dt)18 and random hexamer primers. To confirm the removal of eDNA carryover into eRNA extractions, a qPCR using the short 16S marker (Table 1) was run on each sample post-DNase treatment. Trace eDNA molecules were found to carry over into eRNA extractions following the initial DNase I treatment (from the Zymo *Quick*-DNA/RNA Miniprep Plus Kit), however, eDNA molecules were subsequently removed with a second DNase treatment using the ezDNase enzyme provided with SuperScript IV VILO (ThermoFisher Scientific). After transcription, cDNA was immediately frozen at − 80 °C until further analysis. The cDNA samples are hereafter referred to as eRNA.

PCR reactions were run using 2× Fast Plus EvaGreen qPCR Master Mix (Biotium, California, USA) on an Applied Biosystems QunatStudio Flex 6 Real-Time PCR System. Reactions were 20 µL in volume and each included 10 µL 2× Master Mix, 0.5 µL forward and reverse primers at 10 mM concentration, 6.5 µL diH_2_O, and 2.5 µL of sample template. Cycling began with 10 min at 94 °C followed by 40 cycles of 94 °C for 15 s and 60 °C for 60 s. A negative PCR control was run with each plate of samples. Quantitative standard curves were constructed using diluted PCR products of the four targeted genes (Supplementary Table S1), quantified using Quant-it Picogreen Invitrogen on a Biotek FLX800 plate reader following the instructions manual and log-diluted from 10^6^ to 1 copies/reaction and run-in quadruplicates.

### Statistical analyses

Two replicate tanks (6-mussel replicate B, and 24-mussel replicate B) contained one opened, presumed dead, dreissenid mussel, and thus these replicates were removed from the analysis. All statistical analyses were conducted in R version 3.4.3 software^52^. Differences between initial concentrations (defined as eDNA or eRNA concentration at time 0) were estimated by fitting a linear mixed-effect model using the *R* package ‘lme4’^53^, with log_e_-transformed eDNA/eRNA concentrations at time zero as the response variable. The linear model included categorical fixed effects for gene region, quantitative variable of mussel abundance, and an interaction between abundance and gene region; as well as a nested random effect of samples nested within replicate tanks. The *R* package ‘emmeans’^54^ performed post-hoc Tukey tests to evaluate differences between initial concentrations across mussel abundances, as well as between (a) fragment lengths, (b) gene regions, (c) nucleic acids, and (d) RNA types.

Decay constants were calculated using the time-series change of eDNA/eRNA concentration after mussel removal from each mesocosm. Sampling times for which a qPCR failed to amplify were excluded, as this indicated eDNA/eRNA abundance had decayed below the detection limits (Cycle threshold > 35). Following previous studies, we estimated eDNA/eRNA decay rate by fitting a first-order exponential decay model as follows: C_*t*_ = C_0_e^−k*t^, where C_*t*_ is eDNA/eRNA concentration at time *t* (copies/mL), C_0_ is eDNA/eRNA concentration at time 0, and k is the decay rate constant (per hour)^36,37,55–57^. The decay rates of the exponential decay models were estimated as dC′/dt = − kC′, where C′ = C_*t*_/C_0_ was used to normalize eDNA/eRNA concentration. Previous decay studies have demonstrated biphasic eDNA decay^19,37^, and as such we analyze the log-linear decay rates over the initial 72-h. The decay rates were estimated by fitting a linear mixed-effect model with the values of log_e_ (C_*t*_/C_0_) as the response variable. Sampling time was included as a continuous fixed effect along with categorical fixed effects of gene region, quantitative variable of mussel abundance, and an interaction between abundance and gene region. Two-way interactions between sampling time and mussel abundance and between sampling time and gene region were also included, as well as a nested random effect of samples nested within replicate tanks. The *R* package ‘emmeans’ performed post-hoc Tukey tests to evaluate differences between decay constants across mussel abundances, as well as between (a) fragment lengths, (b) gene regions, (c) nucleic acids, and (d) RNA types. Lastly, the ratio of short:long eDNA fragments (Ratio_S:L_) and eRNA:eDNA (Ratio_R:D_) were calculated for each marker, using the quantified copies/mL across each time point. A linear regression of the log_10_-transformed Ratio_S:L_ and the log_10_-transformed Ratio_R:D_ was used to evaluate differences in degradation over time.

## Supplementary Information

Supplementary Information 1.

Supplementary Information 2.
