# Supplementary material for: Environmental (e)RNA advances the reliability of eDNA by predicting its age
Source: Sci Rep. 2021 Feb 2;11:2769. doi: 10.1038/s41598-021-82205-4 (PMC7854713; doi:10.1038/s41598-021-82205-4)
Supplement: Supplementary file 2 — Supplementary Information 2. [file 41598_2021_82205_MOESM2_ESM.docx]

Supporting Information

Environmental (e)RNA advances the reliability of eDNA by predicting its age

Nathaniel T. Marshall^1^, Henry A.Vanderploeg^2^, and Subba Rao Chaganti^1*^

^1^Cooperative Institute for Great Lakes Research, School for Environment and Sustainability, University of Michigan, 440 Church Street, Ann Arbor, Michigan, USA

^2^National Oceanic and Atmospheric Administration, Great Lakes Environmental Research Laboratory, 4840 S. State Rd, Ann Arbor, Michigan, 48108, USA

Running Head: Degradation rates of *Dreissena* eDNA and eRNA

*corresponding Author: chaganti@umich.edu

**PRIMER TESTING AND DEVELOPMENT**

A literature review searched for possible qPCR primer pairs that are specific to the genus *Dreissena*. As the experimental tanks housed both zebra and quagga mussels, it was important that each identified primer pair amplifies both species. We searched for possible primers that covered both mitochondrial (mt) and nuclear (nu) genes, as well as ribosomal (r)RNA and messenger (m)RNA. We identified three previously designed primer sets that target the mt-rRNA 16S^45^, nu-rRNA 18S^56^, and nu-mRNA H2B^44^ gene regions (Table 1).

All identified mtDNA primer sets appeared to be species-specific, thereby only amplifying zebra or quagga mussel. We therefore developed a *Dreissena* genus-specific mt-mRNA COI primer set (Table 1), capable of amplifying both species. To further analyze differences in degradation of size fragments of eDNA, two primer sets were developed for a long fragment of the mt-rRNA 16S region, and a short fragment of the nu-mRNA H2B region (Table 1). For development of these primers, we downloaded all available sequences of these three gene regions from voucher specimens of both zebra and quagga mussels on the NCBI Genbank database (accessed on November 20th 2019). All sequences were imported into Mega v7 and unique sequences were extracted and aligned using the default settings in the ClustalW alignment tool. A *Dreissena*-specific forward primer was manually developed to amplify 341bp of the mt-rRNA 16S gene when paired with the previously developed reverse primer ^45^. Another *Dreissena*-specific forward primer was manually developed to amplify 75bp of the nu-mRNA H2B gene when paired with the previously developed reverse primer^44^. Finally, a *Dreissena*-specific primer set was developed to amplify 128bp of the mt-mRNA COI gene.

All primer pairs were tested *in vitro* by performing three Real-Time PCR reactions on a six-fold dilution series of genomic DNA from both a zebra and a quagga mussel foot tissue. The presence of a single peak within the melt curve indicated successful amplification of target DNA. Further confidence in the designed primer pairs was provided by Primer-BLAST analysis determining little to no overlap with other freshwater organisms. Finally, quantification standards of the four target regions were developed by amplifying long fragments of each gene region (Table S1). The subsequent PCR product of the four targeted genes was quantified using Quant-it Picogreen Invitrogen on a Biotek FLX800 plate reader following the instructions manual and log-diluted from 10^6^ to 1 copies/reaction and run in quadruplicates for each primer set.

| **Gene** | **Primer Sequence** | **Source** | **Ta** | **Length** |
| --- | --- | --- | --- | --- |
| 16S | **F:** CGCCTGTTTATCAAAAACAT | [57] | 48 | 508 |
|  | **R:** CCGGTCTGAACTCAGATCACGT |  | – |  |
| COI | **F:** GGTCAACAAATCATAAAGATATTGG | [58] | 46 | 709 |
|  | **R:** TAAACTTCAGGGTGACCAAAAAATCA |  | – |  |
| 18S | **F:** CTGGTTGATCCTGCCAG | [59] | 56 | 564 |
|  | **R:** ACCAGACTTGCCCTCC |  | – |  |
| H2B | **F:** AAGTAGGTCAATCCGTTTTCGAG | Current Study | 60 | 460 |
|  | **R:** GCTGGTGTACTTGGTGACAGC |  | – |  |

**Table S1** Primer pairs, annealing temperature (Ta), and the fragment length for the genes regions used to generate standards for the four target qPCR genes.

**REFERENCES**

57. Palumbi, S., Martin, A. & Romano, S. 16S RNA primers. The simple fool's guide to PCR, version 2, 28 (1991).

58. Folmer, O., Black, M., Hoeh, W., Lutz, R. & Vrijenhoek, R. DNA primers for amplification of mitochondrial cytochrome c oxidase subunit I from diverse metazoan invertebrates. *Mol. Mar. Biol. Biotechnol*. **3**, 294–299 (1994).

59. Dı́ez, B. Pedrós-Alió, C.,  Marsh, T. l. & Massana, R. Application of denaturing gradient gel electrophoresis (DGGE) to study the diversity of marine picoeukaryotic assemblages and comparison of DGGE with other molecular techniques. Appl. *Environ. Microbiol*. **67**, 2942–2951 (2001). https://doi.org/10.1128/AEM.67.7.2942-2951.2001

**MODELLING DETAILS AND RESULTS**

***Initial Concentrations at time = 0***

Estimates of the initial eDNA concentrations at time = 0 were obtained using the samples collected after mussel removal from each tank. Differences in eDNA or eRNA concentrations between abundance treatments and gene regions were estimated using a linear mixed-effect model with the log_e_-transformed eDNA or eRNA concentrations as the response variable (y). Mussel abundance (Abundance) and gene region (Gene) were included as fixed effects, and individual tanks (Tank) were included as random effects to correct for differences between tanks. The complete linear mixed-effect model using the R syntax of the ‘lme4’ package is given in equation S1. A conditional R-squared value of 0.9502 was obtained from the model fit. The model summary is given in Table S2 and shows the data used to construct Figure 1 in the manuscript.

Note: Mussel abundance was represented D1- D5 in all the supplementary tables and Figures, Where D1= 2 mussels, D2 = 6 mussels, D3 = 12 mussels, D4 = 24 mussels, D5 = 48 mussels. Further genes were represented as M1 – M10, where M1 = 16S_141 bp DNA, M2 = 16S_341 bp DNA, M3 = COI_128 bp DNA, M4 = 18S_169 bp DNA, M5 = H2B_75 bp DNA, M6 = H2B_250 bp DNA, M7 = 16S_141 bp RNA, M8 = COI_128 bp RNA M9 = 18S_169 bp RNA, M10 = H2B_250 bp RNA.

(S1) model<-lmer(log(y) ~ Gene+Abundance+Gene:Abundance+ (1 | Tank), data)

**Table S2** Summary of the linear mixed-effect model used to evaluate the differences in eDNA or eRNA initial concentration at time = 0 between mussel abundance treatments (Abundance) and gene regions (Gene).

|  | Estimate | SE | t-value |
| --- | --- | --- | --- |
| (Intercept) | 3.906955 | 0.402575 | 9.705 |
| GeneM02 | -0.232441 | 0.421263 | -0.552 |
| GeneM03 | -0.399514 | 0.421263 | -0.948 |
| GeneM04 | 1.898655 | 0.421263 | 4.507 |
| GeneM05 | 2.411652 | 0.421263 | 5.725 |
| GeneM06 | 2.206807 | 0.421263 | 5.239 |
| GeneM07 | 6.529074 | 0.421263 | 15.499 |
| GeneM08 | 3.216031 | 0.421263 | 7.634 |
| GeneM09 | 8.165632 | 0.421263 | 19.384 |
| GeneM10 | -2.879489 | 0.421263 | -6.835 |
| abundance2 | 0.045616 | 0.01567 | 2.911 |
| GeneM02:abundance2 | -0.005347 | 0.016398 | -0.326 |
| GeneM03:abundance2 | -0.006546 | 0.016398 | -0.399 |
| GeneM04:abundance2 | -0.011853 | 0.016398 | -0.723 |
| GeneM05:abundance2 | -0.015881 | 0.016398 | -0.968 |
| GeneM06:abundance2 | -0.010782 | 0.016398 | -0.658 |
| GeneM07:abundance2 | -0.009977 | 0.016398 | -0.608 |
| GeneM08:abundance2 | -0.035252 | 0.016398 | -2.15 |
| GeneM09:abundance2 | -0.016337 | 0.016398 | -0.996 |
| GeneM10:abundance2 | 0.008682 | 0.016398 | 0.529 |

To evaluate differences in initial concentrations at time = 0 between abundance treatments and across gene regions, the R package ‘emmeans’ was used to perform post-hoc Tukey tests of the linear mixed-effects model. Estimated values and comparisons across abundances are shown in Tables S3 and S4, while estimated values and comparisons between gene regions are shown in Tables S5 and S6. Additionally, estimated values and comparisons between gene regions across each mussel abundance are shown in Tables S7 and S8.

**Table S3** Summary of the estimated initial concentration at time = 0 for each mussel abundance treatment (Abundance) from the linear mixed-effect model.

| Abundance | Estimated | SE | df | lower.CL | upper.CL |
| --- | --- | --- | --- | --- | --- |
| 2 | 6.07 | 0.271 | 11 | 5.47 | 6.66 |
| 6 | 6.21 | 0.242 | 11 | 5.68 | 6.74 |
| 12 | 6.42 | 0.209 | 11 | 5.96 | 6.88 |
| 24 | 6.85 | 0.202 | 11 | 6.4 | 7.29 |
| 48 | 7.69 | 0.378 | 11 | 6.86 | 8.52 |

**Table S4** Summary of the post-hoc Tukey test comparisons of the linear mixed-effect model used to evaluate the differences in eDNA or eRNA initial concentration at time = 0 between mussel abundance treatments (Abundance).

| Contrast | Estimate | SE | df | t.ratio | p.value |
| --- | --- | --- | --- | --- | --- |
| 2-6 | -0.141 | 0.0446 | 11 | -3.162 | 0.0562 |
| 2-12 | -0.353 | 0.1116 | 11 | -3.162 | 0.0562 |
| 2-24 | -0.776 | 0.2455 | 11 | -3.162 | 0.0562 |
| 2-48 | -1.623 | 0.5134 | 11 | -3.162 | 0.0562 |
| 6-12 | -0.212 | 0.067 | 11 | -3.162 | 0.0562 |
| 6-24 | -0.635 | 0.2009 | 11 | -3.162 | 0.0562 |
| 6-48 | -1.482 | 0.4687 | 11 | -3.162 | 0.0562 |
| 12-24 | -0.423 | 0.1339 | 11 | -3.162 | 0.0562 |
| 12-48 | -1.27 | 0.4018 | 11 | -3.162 | 0.0562 |
| 24-48 | -0.847 | 0.2679 | 11 | -3.162 | 0.0562 |

**Table S5** Summary of the estimated initial concentration at time = 0 for each gene region (Gene) from the linear mixed-effect model.

| Gene | Estimate | SE | df | lower.CL | upper.CL |
| --- | --- | --- | --- | --- | --- |
| M01 | 4.75 | 0.272 | 38.7 | 4.2 | 5.3 |
| M02 | 4.42 | 0.272 | 38.7 | 3.86 | 4.97 |
| M03 | 4.23 | 0.272 | 38.7 | 3.68 | 4.78 |
| M04 | 6.43 | 0.272 | 38.7 | 5.88 | 6.98 |
| M05 | 6.87 | 0.272 | 38.7 | 6.31 | 7.42 |
| M06 | 6.75 | 0.272 | 38.7 | 6.2 | 7.31 |
| M07 | 11.09 | 0.272 | 38.7 | 10.54 | 11.64 |
| M08 | 7.31 | 0.272 | 38.7 | 6.76 | 7.86 |
| M09 | 12.61 | 0.272 | 38.7 | 12.06 | 13.16 |
| M10 | 2.03 | 0.272 | 38.7 | 1.48 | 2.58 |

**Table S6** Summary of the post-hoc Tukey test comparisons of the linear mixed-effect model used to evaluate the differences in eDNA or eRNA initial concentration at time = 0 between gene regions (Gene).

| Contrast | Estimate | SE | df | t.ratio | p.value |
| --- | --- | --- | --- | --- | --- |
| M01-M02 | 0.331 | 0.285 | 99 | 1.161 | 0.9765 |
| M01-M03 | 0.52 | 0.285 | 99 | 1.824 | 0.7185 |
| M01-M04 | -1.681 | 0.285 | 99 | -5.896 | <.0001 |
| M01-M05 | -2.119 | 0.285 | 99 | -7.435 | <.0001 |
| M01-M06 | -2.008 | 0.285 | 99 | -7.046 | <.0001 |
| M01-M07 | -6.345 | 0.285 | 99 | -22.261 | <.0001 |
| M01-M08 | -2.567 | 0.285 | 99 | -9.007 | <.0001 |
| M01-M09 | -7.865 | 0.285 | 99 | -27.592 | <.0001 |
| M01-M10 | 2.72 | 0.285 | 99 | 9.541 | <.0001 |
| M02-M03 | 0.189 | 0.285 | 99 | 0.664 | 0.9997 |
| M02-M04 | -2.011 | 0.285 | 99 | -7.056 | <.0001 |
| M02-M05 | -2.45 | 0.285 | 99 | -8.596 | <.0001 |
| M02-M06 | -2.339 | 0.285 | 99 | -8.206 | <.0001 |
| M02-M07 | -6.676 | 0.285 | 99 | -23.421 | <.0001 |
| M02-M08 | -2.898 | 0.285 | 99 | -10.167 | <.0001 |
| M02-M09 | -8.196 | 0.285 | 99 | -28.752 | <.0001 |
| M02-M10 | 2.389 | 0.285 | 99 | 8.381 | <.0001 |
| M03-M04 | -2.201 | 0.285 | 99 | -7.72 | <.0001 |
| M03-M05 | -2.639 | 0.285 | 99 | -9.259 | <.0001 |
| M03-M06 | -2.528 | 0.285 | 99 | -8.87 | <.0001 |
| M03-M07 | -6.865 | 0.285 | 99 | -24.085 | <.0001 |
| M03-M08 | -3.087 | 0.285 | 99 | -10.831 | <.0001 |
| M03-M09 | -8.385 | 0.285 | 99 | -29.416 | <.0001 |
| M03-M10 | 2.2 | 0.285 | 99 | 7.717 | <.0001 |
| M04-M05 | -0.439 | 0.285 | 99 | -1.54 | 0.8727 |
| M04-M06 | -0.328 | 0.285 | 99 | -1.15 | 0.9779 |
| M04-M07 | -4.665 | 0.285 | 99 | -16.365 | <.0001 |
| M04-M08 | -0.887 | 0.285 | 99 | -3.111 | 0.0703 |
| M04-M09 | -6.184 | 0.285 | 99 | -21.696 | <.0001 |
| M04-M10 | 4.4 | 0.285 | 99 | 15.437 | <.0001 |
| M05-M06 | 0.111 | 0.285 | 99 | 0.39 | 1 |
| M05-M07 | -4.226 | 0.285 | 99 | -14.826 | <.0001 |
| M05-M08 | -0.448 | 0.285 | 99 | -1.571 | 0.8585 |
| M05-M09 | -5.746 | 0.285 | 99 | -20.156 | <.0001 |
| M05-M10 | 4.839 | 0.285 | 99 | 16.977 | <.0001 |
| M06-M07 | -4.337 | 0.285 | 99 | -15.215 | <.0001 |

**Table S7** Summary of the estimated initial concentration at time = 0 for each gene region (Gene) across each mussel abundance (Abundance) from the linear mixed-effect model.

| Abundance | Gene | Estimated | SE | df | lower.CL | upper.CL |
| --- | --- | --- | --- | --- | --- | --- |
| 2 | M01 | 4 | 0.38 | 38.7 | 3.229 | 4.77 |
|  | M02 | 3.76 | 0.38 | 38.7 | 2.986 | 4.52 |
|  | M03 | 3.59 | 0.38 | 38.7 | 2.817 | 4.35 |
|  | M04 | 5.87 | 0.38 | 38.7 | 5.104 | 6.64 |
|  | M05 | 6.38 | 0.38 | 38.7 | 5.609 | 7.15 |
|  | M06 | 6.18 | 0.38 | 38.7 | 5.414 | 6.95 |
|  | M07 | 10.51 | 0.38 | 38.7 | 9.738 | 11.28 |
|  | M08 | 7.14 | 0.38 | 38.7 | 6.375 | 7.91 |
|  | M09 | 12.13 | 0.38 | 38.7 | 11.362 | 12.9 |
|  | M10 | 1.14 | 0.38 | 38.7 | 0.367 | 1.91 |
| 6 | M01 | 4.18 | 0.339 | 38.7 | 3.494 | 4.87 |
|  | M02 | 3.92 | 0.339 | 38.7 | 3.23 | 4.6 |
|  | M03 | 3.74 | 0.339 | 38.7 | 3.055 | 4.43 |
|  | M04 | 6.01 | 0.339 | 38.7 | 5.322 | 6.69 |
|  | M05 | 6.5 | 0.339 | 38.7 | 5.81 | 7.18 |
|  | M06 | 6.32 | 0.339 | 38.7 | 5.636 | 7.01 |
|  | M07 | 10.65 | 0.339 | 38.7 | 9.963 | 11.34 |
|  | M08 | 7.19 | 0.339 | 38.7 | 6.499 | 7.87 |
|  | M09 | 12.25 | 0.339 | 38.7 | 11.562 | 12.93 |
|  | M10 | 1.35 | 0.339 | 38.7 | 0.667 | 2.04 |
| 12 | M01 | 4.45 | 0.293 | 38.7 | 3.861 | 5.05 |
|  | M02 | 4.16 | 0.293 | 38.7 | 3.565 | 4.75 |
|  | M03 | 3.98 | 0.293 | 38.7 | 3.383 | 4.57 |
|  | M04 | 6.21 | 0.293 | 38.7 | 5.618 | 6.8 |
|  | M05 | 6.68 | 0.293 | 38.7 | 6.082 | 7.27 |
|  | M06 | 6.53 | 0.293 | 38.7 | 5.939 | 7.12 |
|  | M07 | 10.86 | 0.293 | 38.7 | 10.271 | 11.46 |
|  | M08 | 7.25 | 0.293 | 38.7 | 6.654 | 7.84 |
|  | M09 | 12.42 | 0.293 | 38.7 | 11.831 | 13.02 |
|  | M10 | 1.68 | 0.293 | 38.7 | 1.086 | 2.27 |
| 24 | M01 | 5 | 0.284 | 38.7 | 4.428 | 5.58 |
|  | M02 | 4.64 | 0.284 | 38.7 | 4.067 | 5.21 |
|  | M03 | 4.45 | 0.284 | 38.7 | 3.871 | 5.02 |
|  | M04 | 6.62 | 0.284 | 38.7 | 6.042 | 7.19 |
|  | M05 | 7.03 | 0.284 | 38.7 | 6.458 | 7.61 |
|  | M06 | 6.95 | 0.284 | 38.7 | 6.376 | 7.52 |
|  | M07 | 11.29 | 0.284 | 38.7 | 10.717 | 11.87 |
|  | M08 | 7.37 | 0.284 | 38.7 | 6.798 | 7.95 |
|  | M09 | 12.78 | 0.284 | 38.7 | 12.201 | 13.35 |
|  | M10 | 2.33 | 0.284 | 38.7 | 1.757 | 2.9 |
| 48 | M01 | 6.1 | 0.531 | 38.7 | 5.023 | 7.17 |
|  | M02 | 5.61 | 0.531 | 38.7 | 4.534 | 6.68 |
|  | M03 | 5.38 | 0.531 | 38.7 | 4.309 | 6.46 |
|  | M04 | 7.43 | 0.531 | 38.7 | 6.352 | 8.5 |
|  | M05 | 7.75 | 0.531 | 38.7 | 6.672 | 8.82 |
|  | M06 | 7.79 | 0.531 | 38.7 | 6.712 | 8.86 |
|  | M07 | 12.15 | 0.531 | 38.7 | 11.073 | 13.22 |
|  | M08 | 7.62 | 0.531 | 38.7 | 6.547 | 8.69 |
|  | M09 | 13.48 | 0.531 | 38.7 | 12.404 | 14.55 |
|  | M10 | 3.63 | 0.531 | 38.7 | 2.56 | 4.71 |

**Table S8** Summary of the post-hoc Tukey test comparisons of the linear mixed-effect model used to evaluate the differences in eDNA or eRNA initial concentration at time = 0 between gene regions (Gene) across each mussel abundance (Abundance).

| Abundance | contrast | estimate | SE | df | t.ratio | p.value |
| --- | --- | --- | --- | --- | --- | --- |
| 2 | M01-M02 | 0.2431 | 0.398 | 99 | 0.611 | 0.9998 |
|  | M01-M03 | 0.4126 | 0.398 | 99 | 1.037 | 0.9891 |
|  | M01-M04 | -1.8749 | 0.398 | 99 | -4.714 | 0.0003 |
|  | M01-M05 | -2.3799 | 0.398 | 99 | -5.984 | <.0001 |
|  | M01-M06 | -2.1852 | 0.398 | 99 | -5.494 | <.0001 |
|  | M01-M07 | -6.5091 | 0.398 | 99 | -16.366 | <.0001 |
|  | M01-M08 | -3.1455 | 0.398 | 99 | -7.909 | <.0001 |
|  | M01-M09 | -8.133 | 0.398 | 99 | -20.449 | <.0001 |
|  | M01-M10 | 2.8621 | 0.398 | 99 | 7.196 | <.0001 |
|  | M02-M03 | 0.1695 | 0.398 | 99 | 0.426 | 1 |
|  | M02-M04 | -2.1181 | 0.398 | 99 | -5.325 | <.0001 |
|  | M02-M05 | -2.623 | 0.398 | 99 | -6.595 | <.0001 |
|  | M02-M06 | -2.4284 | 0.398 | 99 | -6.106 | <.0001 |
|  | M02-M07 | -6.7523 | 0.398 | 99 | -16.977 | <.0001 |
|  | M02-M08 | -3.3887 | 0.398 | 99 | -8.52 | <.0001 |
|  | M02-M09 | -8.3761 | 0.398 | 99 | -21.06 | <.0001 |
|  | M02-M10 | 2.619 | 0.398 | 99 | 6.585 | <.0001 |
|  | M03-M04 | -2.2876 | 0.398 | 99 | -5.752 | <.0001 |
|  | M03-M05 | -2.7925 | 0.398 | 99 | -7.021 | <.0001 |
|  | M03-M06 | -2.5978 | 0.398 | 99 | -6.532 | <.0001 |
|  | M03-M07 | -6.9217 | 0.398 | 99 | -17.403 | <.0001 |
|  | M03-M08 | -3.5581 | 0.398 | 99 | -8.946 | <.0001 |
|  | M03-M09 | -8.5456 | 0.398 | 99 | -21.486 | <.0001 |
|  | M03-M10 | 2.4495 | 0.398 | 99 | 6.159 | <.0001 |
|  | M04-M05 | -0.5049 | 0.398 | 99 | -1.27 | 0.9581 |
|  | M04-M06 | -0.3103 | 0.398 | 99 | -0.78 | 0.9987 |
|  | M04-M07 | -4.6342 | 0.398 | 99 | -11.652 | <.0001 |
|  | M04-M08 | -1.2706 | 0.398 | 99 | -3.195 | 0.0562 |
|  | M04-M09 | -6.258 | 0.398 | 99 | -15.734 | <.0001 |
|  | M04-M10 | 4.7371 | 0.398 | 99 | 11.91 | <.0001 |
|  | M05-M06 | 0.1946 | 0.398 | 99 | 0.489 | 1 |
|  | M05-M07 | -4.1292 | 0.398 | 99 | -10.382 | <.0001 |
|  | M05-M08 | -0.7656 | 0.398 | 99 | -1.925 | 0.6522 |
|  | M05-M09 | -5.7531 | 0.398 | 99 | -14.465 | <.0001 |
|  | M05-M10 | 5.242 | 0.398 | 99 | 13.18 | <.0001 |
|  | M06-M07 | -4.3239 | 0.398 | 99 | -10.871 | <.0001 |
|  | M06-M08 | -0.9603 | 0.398 | 99 | -2.414 | 0.3289 |
|  | M06-M09 | -5.9477 | 0.398 | 99 | -14.954 | <.0001 |
|  | M06-M10 | 5.0474 | 0.398 | 99 | 12.691 | <.0001 |
|  | M07-M08 | 3.3636 | 0.398 | 99 | 8.457 | <.0001 |
|  | M07-M09 | -1.6238 | 0.398 | 99 | -4.083 | 0.0035 |
|  | M07-M10 | 9.3712 | 0.398 | 99 | 23.562 | <.0001 |
|  | M08-M09 | -4.9874 | 0.398 | 99 | -12.54 | <.0001 |
|  | M08-M10 | 6.0077 | 0.398 | 99 | 15.105 | <.0001 |
|  | M09-M10 | 10.9951 | 0.398 | 99 | 27.645 | <.0001 |
| 6 | M01-M02 | 0.2645 | 0.355 | 99 | 0.745 | 0.9991 |
|  | M01-M03 | 0.4388 | 0.355 | 99 | 1.236 | 0.9646 |
|  | M01-M04 | -1.8275 | 0.355 | 99 | -5.147 | 0.0001 |
|  | M01-M05 | -2.3164 | 0.355 | 99 | -6.523 | <.0001 |
|  | M01-M06 | -2.1421 | 0.355 | 99 | -6.033 | <.0001 |
|  | M01-M07 | -6.4692 | 0.355 | 99 | -18.219 | <.0001 |
|  | M01-M08 | -3.0045 | 0.355 | 99 | -8.461 | <.0001 |
|  | M01-M09 | -8.0676 | 0.355 | 99 | -22.72 | <.0001 |
|  | M01-M10 | 2.8274 | 0.355 | 99 | 7.963 | <.0001 |
|  | M02-M03 | 0.1743 | 0.355 | 99 | 0.491 | 1 |
|  | M02-M04 | -2.0921 | 0.355 | 99 | -5.892 | <.0001 |
|  | M02-M05 | -2.5809 | 0.355 | 99 | -7.268 | <.0001 |
|  | M02-M06 | -2.4066 | 0.355 | 99 | -6.778 | <.0001 |
|  | M02-M07 | -6.7337 | 0.355 | 99 | -18.964 | <.0001 |
|  | M02-M08 | -3.269 | 0.355 | 99 | -9.206 | <.0001 |
|  | M02-M09 | -8.3321 | 0.355 | 99 | -23.465 | <.0001 |
|  | M02-M10 | 2.5629 | 0.355 | 99 | 7.218 | <.0001 |
|  | M03-M04 | -2.2663 | 0.355 | 99 | -6.383 | <.0001 |
|  | M03-M05 | -2.7552 | 0.355 | 99 | -7.759 | <.0001 |
|  | M03-M06 | -2.5809 | 0.355 | 99 | -7.268 | <.0001 |
|  | M03-M07 | -6.908 | 0.355 | 99 | -19.454 | <.0001 |
|  | M03-M08 | -3.4433 | 0.355 | 99 | -9.697 | <.0001 |
|  | M03-M09 | -8.5064 | 0.355 | 99 | -23.956 | <.0001 |
|  | M03-M10 | 2.3886 | 0.355 | 99 | 6.727 | <.0001 |
|  | M04-M05 | -0.4888 | 0.355 | 99 | -1.377 | 0.9315 |
|  | M04-M06 | -0.3146 | 0.355 | 99 | -0.886 | 0.9966 |
|  | M04-M07 | -4.6417 | 0.355 | 99 | -13.072 | <.0001 |
|  | M04-M08 | -1.177 | 0.355 | 99 | -3.315 | 0.0402 |
|  | M04-M09 | -6.2401 | 0.355 | 99 | -17.573 | <.0001 |
|  | M04-M10 | 4.6549 | 0.355 | 99 | 13.109 | <.0001 |
|  | M05-M06 | 0.1743 | 0.355 | 99 | 0.491 | 1 |
|  | M05-M07 | -4.1528 | 0.355 | 99 | -11.695 | <.0001 |
|  | M05-M08 | -0.6882 | 0.355 | 99 | -1.938 | 0.6435 |
|  | M05-M09 | -5.7512 | 0.355 | 99 | -16.197 | <.0001 |
|  | M05-M10 | 5.1438 | 0.355 | 99 | 14.486 | <.0001 |
|  | M06-M07 | -4.3271 | 0.355 | 99 | -12.186 | <.0001 |
|  | M06-M08 | -0.8624 | 0.355 | 99 | -2.429 | 0.3207 |
|  | M06-M09 | -5.9255 | 0.355 | 99 | -16.688 | <.0001 |
|  | M06-M10 | 4.9695 | 0.355 | 99 | 13.995 | <.0001 |
|  | M07-M08 | 3.4647 | 0.355 | 99 | 9.757 | <.0001 |
|  | M07-M09 | -1.5984 | 0.355 | 99 | -4.501 | 0.0007 |
|  | M07-M10 | 9.2966 | 0.355 | 99 | 26.181 | <.0001 |
|  | M08-M09 | -5.0631 | 0.355 | 99 | -14.259 | <.0001 |
|  | M08-M10 | 5.8319 | 0.355 | 99 | 16.424 | <.0001 |
|  | M09-M10 | 10.895 | 0.355 | 99 | 30.683 | <.0001 |
| 12 | M01-M02 | 0.2966 | 0.307 | 99 | 0.967 | 0.9934 |
|  | M01-M03 | 0.4781 | 0.307 | 99 | 1.559 | 0.8643 |
|  | M01-M04 | -1.7564 | 0.307 | 99 | -5.727 | <.0001 |
|  | M01-M05 | -2.2211 | 0.307 | 99 | -7.242 | <.0001 |
|  | M01-M06 | -2.0774 | 0.307 | 99 | -6.773 | <.0001 |
|  | M01-M07 | -6.4093 | 0.307 | 99 | -20.897 | <.0001 |
|  | M01-M08 | -2.793 | 0.307 | 99 | -9.107 | <.0001 |
|  | M01-M09 | -7.9696 | 0.307 | 99 | -25.985 | <.0001 |
|  | M01-M10 | 2.7753 | 0.307 | 99 | 9.049 | <.0001 |
|  | M02-M03 | 0.1815 | 0.307 | 99 | 0.592 | 0.9999 |
|  | M02-M04 | -2.053 | 0.307 | 99 | -6.694 | <.0001 |
|  | M02-M05 | -2.5177 | 0.307 | 99 | -8.209 | <.0001 |
|  | M02-M06 | -2.374 | 0.307 | 99 | -7.74 | <.0001 |
|  | M02-M07 | -6.7059 | 0.307 | 99 | -21.865 | <.0001 |
|  | M02-M08 | -3.0896 | 0.307 | 99 | -10.074 | <.0001 |
|  | M02-M09 | -8.2662 | 0.307 | 99 | -26.952 | <.0001 |
|  | M02-M10 | 2.4787 | 0.307 | 99 | 8.082 | <.0001 |
|  | M03-M04 | -2.2345 | 0.307 | 99 | -7.285 | <.0001 |
|  | M03-M05 | -2.6992 | 0.307 | 99 | -8.8 | <.0001 |
|  | M03-M06 | -2.5555 | 0.307 | 99 | -8.332 | <.0001 |
|  | M03-M07 | -6.8874 | 0.307 | 99 | -22.456 | <.0001 |
|  | M03-M08 | -3.2711 | 0.307 | 99 | -10.665 | <.0001 |
|  | M03-M09 | -8.4477 | 0.307 | 99 | -27.543 | <.0001 |
|  | M03-M10 | 2.2972 | 0.307 | 99 | 7.49 | <.0001 |
|  | M04-M05 | -0.4647 | 0.307 | 99 | -1.515 | 0.883 |
|  | M04-M06 | -0.321 | 0.307 | 99 | -1.047 | 0.9884 |
|  | M04-M07 | -4.6529 | 0.307 | 99 | -15.171 | <.0001 |
|  | M04-M08 | -1.0366 | 0.307 | 99 | -3.38 | 0.0333 |
|  | M04-M09 | -6.2132 | 0.307 | 99 | -20.258 | <.0001 |
|  | M04-M10 | 4.5317 | 0.307 | 99 | 14.776 | <.0001 |
|  | M05-M06 | 0.1437 | 0.307 | 99 | 0.468 | 1 |
|  | M05-M07 | -4.1883 | 0.307 | 99 | -13.656 | <.0001 |
|  | M05-M08 | -0.5719 | 0.307 | 99 | -1.865 | 0.6923 |
|  | M05-M09 | -5.7485 | 0.307 | 99 | -18.743 | <.0001 |
|  | M05-M10 | 4.9964 | 0.307 | 99 | 16.291 | <.0001 |
|  | M06-M07 | -4.3319 | 0.307 | 99 | -14.124 | <.0001 |
|  | M06-M08 | -0.7156 | 0.307 | 99 | -2.333 | 0.3776 |
|  | M06-M09 | -5.8922 | 0.307 | 99 | -19.211 | <.0001 |
|  | M06-M10 | 4.8527 | 0.307 | 99 | 15.822 | <.0001 |
|  | M07-M08 | 3.6163 | 0.307 | 99 | 11.791 | <.0001 |
|  | M07-M09 | -1.5602 | 0.307 | 99 | -5.087 | 0.0001 |
|  | M07-M10 | 9.1846 | 0.307 | 99 | 29.946 | <.0001 |
|  | M08-M09 | -5.1766 | 0.307 | 99 | -16.878 | <.0001 |
|  | M08-M10 | 5.5683 | 0.307 | 99 | 18.155 | <.0001 |
|  | M09-M10 | 10.7449 | 0.307 | 99 | 35.033 | <.0001 |
| 24 | M01-M02 | 0.3608 | 0.297 | 99 | 1.215 | 0.9682 |
|  | M01-M03 | 0.5566 | 0.297 | 99 | 1.875 | 0.6854 |
|  | M01-M04 | -1.6142 | 0.297 | 99 | -5.438 | <.0001 |
|  | M01-M05 | -2.0305 | 0.297 | 99 | -6.841 | <.0001 |
|  | M01-M06 | -1.948 | 0.297 | 99 | -6.563 | <.0001 |
|  | M01-M07 | -6.2896 | 0.297 | 99 | -21.189 | <.0001 |
|  | M01-M08 | -2.37 | 0.297 | 99 | -7.984 | <.0001 |
|  | M01-M09 | -7.7735 | 0.297 | 99 | -26.188 | <.0001 |
|  | M01-M10 | 2.6711 | 0.297 | 99 | 8.999 | <.0001 |
|  | M02-M03 | 0.1959 | 0.297 | 99 | 0.66 | 0.9997 |
|  | M02-M04 | -1.975 | 0.297 | 99 | -6.653 | <.0001 |
|  | M02-M05 | -2.3913 | 0.297 | 99 | -8.056 | <.0001 |
|  | M02-M06 | -2.3088 | 0.297 | 99 | -7.778 | <.0001 |
|  | M02-M07 | -6.6504 | 0.297 | 99 | -22.404 | <.0001 |
|  | M02-M08 | -2.7307 | 0.297 | 99 | -9.2 | <.0001 |
|  | M02-M09 | -8.1343 | 0.297 | 99 | -27.403 | <.0001 |
|  | M02-M10 | 2.3104 | 0.297 | 99 | 7.783 | <.0001 |
|  | M03-M04 | -2.1708 | 0.297 | 99 | -7.313 | <.0001 |
|  | M03-M05 | -2.5871 | 0.297 | 99 | -8.716 | <.0001 |
|  | M03-M06 | -2.5047 | 0.297 | 99 | -8.438 | <.0001 |
|  | M03-M07 | -6.8462 | 0.297 | 99 | -23.064 | <.0001 |
|  | M03-M08 | -2.9266 | 0.297 | 99 | -9.859 | <.0001 |
|  | M03-M09 | -8.3302 | 0.297 | 99 | -28.063 | <.0001 |
|  | M03-M10 | 2.1145 | 0.297 | 99 | 7.123 | <.0001 |
|  | M04-M05 | -0.4163 | 0.297 | 99 | -1.403 | 0.9237 |
|  | M04-M06 | -0.3338 | 0.297 | 99 | -1.125 | 0.981 |
|  | M04-M07 | -4.6754 | 0.297 | 99 | -15.751 | <.0001 |
|  | M04-M08 | -0.7558 | 0.297 | 99 | -2.546 | 0.2577 |
|  | M04-M09 | -6.1594 | 0.297 | 99 | -20.75 | <.0001 |
|  | M04-M10 | 4.2853 | 0.297 | 99 | 14.437 | <.0001 |
|  | M05-M06 | 0.0825 | 0.297 | 99 | 0.278 | 1 |
|  | M05-M07 | -4.2591 | 0.297 | 99 | -14.348 | <.0001 |
|  | M05-M08 | -0.3395 | 0.297 | 99 | -1.144 | 0.9787 |
|  | M05-M09 | -5.743 | 0.297 | 99 | -19.348 | <.0001 |
|  | M05-M10 | 4.7016 | 0.297 | 99 | 15.839 | <.0001 |
|  | M06-M07 | -4.3416 | 0.297 | 99 | -14.626 | <.0001 |
|  | M06-M08 | -0.422 | 0.297 | 99 | -1.422 | 0.9176 |
|  | M06-M09 | -5.8255 | 0.297 | 99 | -19.625 | <.0001 |
|  | M06-M10 | 4.6191 | 0.297 | 99 | 15.561 | <.0001 |
|  | M07-M08 | 3.9196 | 0.297 | 99 | 13.205 | <.0001 |
|  | M07-M09 | -1.4839 | 0.297 | 99 | -4.999 | 0.0001 |
|  | M07-M10 | 8.9607 | 0.297 | 99 | 30.188 | <.0001 |
|  | M08-M09 | -5.4036 | 0.297 | 99 | -18.204 | <.0001 |
|  | M08-M10 | 5.0411 | 0.297 | 99 | 16.983 | <.0001 |
|  | M09-M10 | 10.4447 | 0.297 | 99 | 35.187 | <.0001 |
| 48 | M01-M02 | 0.4891 | 0.555 | 99 | 0.881 | 0.9967 |
|  | M01-M03 | 0.7137 | 0.555 | 99 | 1.285 | 0.9548 |
|  | M01-M04 | -1.3297 | 0.555 | 99 | -2.394 | 0.3408 |
|  | M01-M05 | -1.6494 | 0.555 | 99 | -2.969 | 0.1009 |
|  | M01-M06 | -1.6893 | 0.555 | 99 | -3.041 | 0.0842 |
|  | M01-M07 | -6.0502 | 0.555 | 99 | -10.893 | <.0001 |
|  | M01-M08 | -1.5239 | 0.555 | 99 | -2.744 | 0.1712 |
|  | M01-M09 | -7.3815 | 0.555 | 99 | -13.289 | <.0001 |
|  | M01-M10 | 2.4627 | 0.555 | 99 | 4.434 | 0.001 |
|  | M02-M03 | 0.2247 | 0.555 | 99 | 0.404 | 1 |
|  | M02-M04 | -1.8188 | 0.555 | 99 | -3.275 | 0.045 |
|  | M02-M05 | -2.1385 | 0.555 | 99 | -3.85 | 0.0077 |
|  | M02-M06 | -2.1783 | 0.555 | 99 | -3.922 | 0.006 |
|  | M02-M07 | -6.5392 | 0.555 | 99 | -11.773 | <.0001 |
|  | M02-M08 | -2.013 | 0.555 | 99 | -3.624 | 0.0159 |
|  | M02-M09 | -7.8705 | 0.555 | 99 | -14.17 | <.0001 |
|  | M02-M10 | 1.9737 | 0.555 | 99 | 3.553 | 0.0198 |
|  | M03-M04 | -2.0435 | 0.555 | 99 | -3.679 | 0.0134 |
|  | M03-M05 | -2.3631 | 0.555 | 99 | -4.254 | 0.0019 |
|  | M03-M06 | -2.403 | 0.555 | 99 | -4.326 | 0.0014 |
|  | M03-M07 | -6.7639 | 0.555 | 99 | -12.178 | <.0001 |
|  | M03-M08 | -2.2377 | 0.555 | 99 | -4.029 | 0.0042 |
|  | M03-M09 | -8.0952 | 0.555 | 99 | -14.574 | <.0001 |
|  | M03-M10 | 1.749 | 0.555 | 99 | 3.149 | 0.0636 |
|  | M04-M05 | -0.3196 | 0.555 | 99 | -0.575 | 0.9999 |
|  | M04-M06 | -0.3595 | 0.555 | 99 | -0.647 | 0.9997 |
|  | M04-M07 | -4.7204 | 0.555 | 99 | -8.499 | <.0001 |
|  | M04-M08 | -0.1942 | 0.555 | 99 | -0.35 | 1 |
|  | M04-M09 | -6.0517 | 0.555 | 99 | -10.895 | <.0001 |
|  | M04-M10 | 3.7925 | 0.555 | 99 | 6.828 | <.0001 |
|  | M05-M06 | -0.0399 | 0.555 | 99 | -0.072 | 1 |
|  | M05-M07 | -4.4008 | 0.555 | 99 | -7.923 | <.0001 |
|  | M05-M08 | 0.1254 | 0.555 | 99 | 0.226 | 1 |
|  | M05-M09 | -5.7321 | 0.555 | 99 | -10.32 | <.0001 |
|  | M05-M10 | 4.1121 | 0.555 | 99 | 7.403 | <.0001 |
|  | M06-M07 | -4.3609 | 0.555 | 99 | -7.851 | <.0001 |
|  | M06-M08 | 0.1653 | 0.555 | 99 | 0.298 | 1 |
|  | M06-M09 | -5.6922 | 0.555 | 99 | -10.248 | <.0001 |
|  | M06-M10 | 4.152 | 0.555 | 99 | 7.475 | <.0001 |
|  | M07-M08 | 4.5262 | 0.555 | 99 | 8.149 | <.0001 |
|  | M07-M09 | -1.3313 | 0.555 | 99 | -2.397 | 0.3391 |
|  | M07-M10 | 8.5129 | 0.555 | 99 | 15.326 | <.0001 |
|  | M08-M09 | -5.8575 | 0.555 | 99 | -10.546 | <.0001 |
|  | M08-M10 | 3.9867 | 0.555 | 99 | 7.178 | <.0001 |
|  | M09-M10 | 9.8442 | 0.555 | 99 | 17.723 | <.0001 |


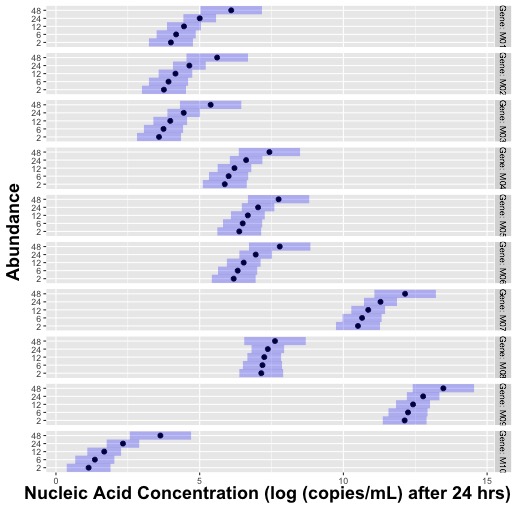


**Figure S1** Relationship between mussel abundance and eDNA or eRNA initial concentration at time = 0 for each of the target gene regions estimated from the linear mixed-effects model.

To further evaluate differences in initial concentrations at time = 0 between abundance treatments, a power function was modeled using the ‘nls’ function in R as shown in equation S2. The results of these models for each gene region is displayed in figure S1.

(S2) model<-nls(y ~ b*Abundance^z, data=data, start = c(b = 4, z = 0.1))

**Figure S2** Relationship between mussel abundance and eDNA or eRNA initial concentration at time = 0 for each of the target gene regions.

***Decay rates***

Estimates of the eDNA and eRNA decay constants were calculated using the time-series change of concentrations after mussel removal from each mesocosm. Differences in decay constants between abundance treatments and gene regions were estimated using a linear mixed-effect model with the values of log_e_(C*_t_*/C_0_) as the response variable (y) across the first 72 hours. Sampling time (Time), mussel abundance (Abundance), and gene region (Gene) were included as fixed effects, along with two-way interactions between sampling time and mussel abundance (Abundance:Time), sampling time and gene region (Gene:Time), and gene region across mussel abundance over time (Gene:Abundance:Time). The complete linear mixed-effect model is given in equation S3. A conditional R-squared value of 0.8810 was obtained from the model fit. The model summary is given in Table S9 and includes the data used to construct Figure 2 in the manuscript.

(S3) model<-lmer(log(y) ~ 0 + Gene + Abundance + Time + Gene:Time + Abundance:Time + Gene:Abundance:Time + (1 | Tank), data)

**Table S9** Summary of the linear mixed-effect model used to evaluate the differences in eDNA or eRNA decay constant between mussel abundance treatments (Abundance) and gene regions (Gene).

|  | Estimate | SE | t-value |
| --- | --- | --- | --- |
| GeneM1 | -4.19E-02 | 1.79E-01 | -0.233 |
| GeneM2 | 2.33E-02 | 1.79E-01 | 0.13 |
| GeneM3 | -7.40E-02 | 1.79E-01 | -0.413 |
| GeneM4 | 3.95E-02 | 1.80E-01 | 0.22 |
| GeneM5 | 1.08E-01 | 1.79E-01 | 0.6 |
| GeneM6 | 3.79E-02 | 1.80E-01 | 0.211 |
| GeneM7 | 1.74E-01 | 1.79E-01 | 0.969 |
| GeneM8 | 1.30E-01 | 1.79E-01 | 0.726 |
| GeneM9 | -1.30E-01 | 1.79E-01 | -0.725 |
| abun | -5.36E-03 | 6.35E-03 | -0.844 |
| Time | -1.49E-02 | 3.19E-03 | -4.663 |
| GeneM2:Time | -3.68E-03 | 4.41E-03 | -0.835 |
| GeneM3:Time | 3.06E-03 | 4.42E-03 | 0.693 |
| GeneM4:Time | -8.36E-03 | 4.47E-03 | -1.871 |
| GeneM5:Time | -8.08E-03 | 4.41E-03 | -1.832 |
| GeneM6:Time | -9.06E-03 | 4.47E-03 | -2.029 |
| GeneM7:Time | -1.18E-02 | 4.41E-03 | -2.664 |
| GeneM8:Time | -1.96E-02 | 4.41E-03 | -4.45 |
| GeneM9:Time | -3.38E-02 | 4.41E-03 | -7.655 |
| abun:Time | -1.27E-03 | 1.09E-04 | -11.62 |
| GeneM2:abun:Time | -2.89E-05 | 1.46E-04 | -0.198 |
| GeneM3:abun:Time | -2.83E-04 | 1.52E-04 | -1.868 |
| GeneM4:abun:Time | -2.38E-05 | 1.73E-04 | -0.137 |
| GeneM5:abun:Time | -1.35E-05 | 1.46E-04 | -0.093 |
| GeneM6:abun:Time | -6.53E-05 | 1.73E-04 | -0.377 |
| GeneM7:abun:Time | -3.63E-04 | 1.46E-04 | -2.489 |
| GeneM8:abun:Time | -1.55E-04 | 1.46E-04 | -1.065 |
| GeneM9:abun:Time | -1.09E-04 | 1.46E-04 | -0.749 |

Next, to evaluate differences in decay constants between abundance treatments and across gene regions, the emtrends command implemented in the R package ‘emmeans’ was used to perform post-hoc Tukey tests of the linear mixed-effects model. Estimated values and comparisons across abundances are shown in Tables S10 and S11, while estimated values and comparisons between gene regions are shown in Tables S12 and S13. Additionally, estimated values and comparisons between gene regions across within each mussel abundance are shown in Tables S14 and S15.

**Table S10** Summary of the estimated decay constant (k) for each mussel abundance treatment (Abundance) from the linear mixed-effect model.

| Abundance | Estimate | SE | df | lower.CL | upper.CL |
| --- | --- | --- | --- | --- | --- |
| 2 | -0.0278 | 0.00117 | 758 | -0.0301 | -0.0255 |
| 6 | -0.0333 | 0.001033 | 758 | -0.0354 | -0.0313 |
| 12 | -0.0416 | 0.000887 | 758 | -0.0434 | -0.0399 |
| 24 | -0.0583 | 0.000917 | 758 | -0.0601 | -0.0565 |
| 48 | -0.0915 | 0.001848 | 759 | -0.0951 | -0.0879 |

**Table S11** Summary of the post-hoc Tukey test comparisons of the linear mixed-effect model used to evaluate the differences in eDNA or eRNA decay constant (k) between mussel abundance treatments (Abundance).

| Contrast | Estimate | SE | df | t.ratio | p.value |
| --- | --- | --- | --- | --- | --- |
| 2-6 | 0.00554 | 0.000214 | 759 | 25.942 | <.0001 |
| 2-12 | 0.01385 | 0.000534 | 759 | 25.942 | <.0001 |
| 2-24 | 0.03048 | 0.001175 | 759 | 25.942 | <.0001 |
| 2-48 | 0.06373 | 0.002457 | 759 | 25.942 | <.0001 |
| 6-12 | 0.00831 | 0.00032 | 759 | 25.942 | <.0001 |
| 6-24 | 0.02494 | 0.000961 | 759 | 25.942 | <.0001 |
| 6-48 | 0.05819 | 0.002243 | 759 | 25.942 | <.0001 |
| 12-24 | 0.01663 | 0.000641 | 759 | 25.942 | <.0001 |
| 12-48 | 0.04988 | 0.001923 | 759 | 25.942 | <.0001 |
| 24-48 | 0.03325 | 0.001282 | 759 | 25.942 | <.0001 |

**Table S12** Summary of the estimated decay constant (k) for each gene region (Gene) from the linear mixed-effect model.

| Gene | Estimate | SE | df | lower.CL | upper.CL |
| --- | --- | --- | --- | --- | --- |
| M1 | -0.0378 | 0.0025 | 758 | -0.0427 | -0.0329 |
| M2 | -0.0420 | 0.0025 | 758 | -0.0469 | -0.0371 |
| M3 | -0.0398 | 0.0025 | 758 | -0.0448 | -0.0349 |
| M4 | -0.0466 | 0.0027 | 758 | -0.0518 | -0.0413 |
| M5 | -0.0461 | 0.0025 | 758 | -0.051 | -0.0413 |
| M6 | -0.0480 | 0.0027 | 758 | -0.0533 | -0.0428 |
| M7 | -0.0561 | 0.0025 | 758 | -0.061 | -0.0512 |
| M8 | -0.0602 | 0.0025 | 758 | -0.0651 | -0.0554 |
| M9 | -0.0735 | 0.0025 | 758 | -0.0784 | -0.0687 |

**Table S13** Summary of the post-hoc Tukey test comparisons of the linear mixed-effect model used to evaluate the differences in eDNA or eRNA decay constant (k) between gene regions (Gene).

| Contrast | Estimate | SE | df | t.ratio | p.value |
| --- | --- | --- | --- | --- | --- |
| M01-M02 | 0.004202 | 0.0035 | 758 | 1.2 | 0.9564 |
| M01-M03 | 0.002054 | 3.54E-03 | 758 | 0.581 | 0.9997 |
| M01-M04 | 0.008788 | 3.65E-03 | 758 | 2.409 | 0.2804 |
| M01-M05 | 0.008323 | 3.50E-03 | 758 | 2.377 | 0.2982 |
| M01-M06 | 0.010242 | 3.65E-03 | 758 | 2.807 | 0.1148 |
| M01-M07 | 0.018298 | 3.50E-03 | 758 | 5.226 | <.0001 |
| M01-M08 | 0.022427 | 3.50E-03 | 758 | 6.405 | <.0001 |
| M01-M09 | 0.035731 | 3.50E-03 | 758 | 10.204 | <.0001 |
| M02-M03 | -0.002148 | 3.54E-03 | 758 | -0.607 | 0.9996 |
| M02-M04 | 0.004586 | 3.65E-03 | 758 | 1.257 | 0.9431 |
| M02-M05 | 0.00412 | 3.50E-03 | 758 | 1.177 | 0.9612 |
| M02-M06 | 0.00604 | 3.65E-03 | 758 | 1.655 | 0.7734 |
| M02-M07 | 0.014095 | 3.50E-03 | 758 | 4.025 | 0.002 |
| M02-M08 | 0.018224 | 3.50E-03 | 758 | 5.205 | <.0001 |
| M02-M09 | 0.031528 | 3.50E-03 | 758 | 9.004 | <.0001 |
| M03-M04 | 0.006734 | 3.68E-03 | 758 | 1.83 | 0.6622 |
| M03-M05 | 0.006268 | 3.54E-03 | 758 | 1.772 | 0.7006 |
| M03-M06 | 0.008188 | 3.68E-03 | 758 | 2.225 | 0.3904 |
| M03-M07 | 0.016243 | 3.54E-03 | 758 | 4.592 | 0.0002 |
| M03-M08 | 0.020373 | 3.54E-03 | 758 | 5.76 | <.0001 |
| M03-M09 | 0.033676 | 3.54E-03 | 758 | 9.521 | <.0001 |
| M04-M05 | -0.000466 | 3.65E-03 | 758 | -0.128 | 1 |
| M04-M06 | 0.001454 | 3.78E-03 | 758 | 0.385 | 1 |
| M04-M07 | 0.009509 | 3.65E-03 | 758 | 2.606 | 0.1855 |
| M04-M08 | 0.013638 | 3.65E-03 | 758 | 3.738 | 0.0062 |
| M04-M09 | 0.026942 | 3.65E-03 | 758 | 7.385 | <.0001 |
| M05-M06 | 0.00192 | 3.65E-03 | 758 | 0.526 | 0.9999 |
| M05-M07 | 0.009975 | 3.50E-03 | 758 | 2.849 | 0.1033 |
| M05-M08 | 0.014104 | 3.50E-03 | 758 | 4.028 | 0.002 |
| M05-M09 | 0.027408 | 3.50E-03 | 758 | 7.828 | <.0001 |
| M06-M07 | 0.008055 | 3.65E-03 | 758 | 2.208 | 0.4014 |
| M06-M08 | 0.012185 | 3.65E-03 | 758 | 3.34 | 0.0246 |
| M06-M09 | 0.025488 | 3.65E-03 | 758 | 6.987 | <.0001 |
| M07-M08 | 0.004129 | 3.50E-03 | 758 | 1.179 | 0.9607 |
| M07-M09 | 0.017433 | 3.50E-03 | 758 | 4.979 | <.0001 |
| M08-M09 | 0.013304 | 3.50E-03 | 758 | 3.799 | 0.0049 |

**Table S14** Summary of the estimated decay constant for each gene region (Gene) across each mussel abundance (Abundance) from the linear mixed-effect model.

| Abundance | Gene | Estimated | SE | df | lower.CL | upper.CL |
| --- | --- | --- | --- | --- | --- | --- |
| 2 | M01 | -0.0174 | 0.00306 | 758 | -0.0234 | -0.0114 |
|  | M02 | -0.0211 | 0.00306 | 758 | -0.0271 | -0.0151 |
|  | M03 | -0.0149 | 0.00306 | 758 | -0.0209 | -0.0089 |
|  | M04 | -0.0258 | 0.00311 | 758 | -0.0319 | -0.0197 |
|  | M05 | -0.0255 | 0.00306 | 758 | -0.0315 | -0.0195 |
|  | M06 | -0.0266 | 0.00311 | 758 | -0.0327 | -0.0205 |
|  | M07 | -0.0299 | 0.00306 | 758 | -0.0359 | -0.0239 |
|  | M08 | -0.0373 | 0.00306 | 758 | -0.0433 | -0.0313 |
|  | M09 | -0.0514 | 0.00306 | 758 | -0.0574 | -0.0454 |
| 6 | M01 | -0.0225 | 0.00282 | 758 | -0.028 | -0.0169 |
|  | M02 | -0.0263 | 0.00282 | 758 | -0.0319 | -0.0208 |
|  | M03 | -0.0211 | 0.00282 | 758 | -0.0267 | -0.0156 |
|  | M04 | -0.031 | 0.00283 | 758 | -0.0366 | -0.0254 |
|  | M05 | -0.0306 | 0.00282 | 758 | -0.0362 | -0.0251 |
|  | M06 | -0.0319 | 0.00283 | 758 | -0.0375 | -0.0264 |
|  | M07 | -0.0364 | 0.00282 | 758 | -0.042 | -0.0309 |
|  | M08 | -0.043 | 0.00282 | 758 | -0.0486 | -0.0375 |
|  | M09 | -0.0569 | 0.00282 | 758 | -0.0624 | -0.0514 |
| 12 | M01 | -0.0301 | 0.00257 | 758 | -0.0352 | -0.0251 |
|  | M02 | -0.0341 | 0.00257 | 758 | -0.0392 | -0.0291 |
|  | M03 | -0.0304 | 0.00258 | 758 | -0.0355 | -0.0254 |
|  | M04 | -0.0388 | 0.00261 | 758 | -0.0439 | -0.0336 |
|  | M05 | -0.0383 | 0.00257 | 758 | -0.0434 | -0.0333 |
|  | M06 | -0.04 | 0.00261 | 758 | -0.0451 | -0.0348 |
|  | M07 | -0.0462 | 0.00257 | 758 | -0.0513 | -0.0412 |
|  | M08 | -0.0516 | 0.00257 | 758 | -0.0566 | -0.0465 |
|  | M09 | -0.0652 | 0.00257 | 758 | -0.0702 | -0.0601 |
| 24 | M01 | -0.0453 | 0.00255 | 758 | -0.0504 | -0.0403 |
|  | M02 | -0.0497 | 0.00255 | 758 | -0.0547 | -0.0447 |
|  | M03 | -0.0491 | 0.00266 | 758 | -0.0543 | -0.0439 |
|  | M04 | -0.0543 | 0.003 | 759 | -0.0602 | -0.0484 |
|  | M05 | -0.0537 | 0.00255 | 758 | -0.0588 | -0.0487 |
|  | M06 | -0.056 | 0.003 | 759 | -0.0619 | -0.0501 |
|  | M07 | -0.0658 | 0.00255 | 758 | -0.0708 | -0.0608 |
|  | M08 | -0.0687 | 0.00255 | 758 | -0.0737 | -0.0637 |
|  | M09 | -0.0817 | 0.00255 | 758 | -0.0867 | -0.0767 |
| 48 | M01 | -0.0758 | 0.00407 | 758 | -0.0838 | -0.0678 |
|  | M02 | -0.0809 | 0.00407 | 758 | -0.0889 | -0.0729 |
|  | M03 | -0.0864 | 0.00446 | 758 | -0.0951 | -0.0776 |
|  | M04 | -0.0853 | 0.00563 | 759 | -0.0964 | -0.0743 |
|  | M05 | -0.0845 | 0.00407 | 758 | -0.0925 | -0.0765 |
|  | M06 | -0.088 | 0.00563 | 759 | -0.0991 | -0.077 |
|  | M07 | -0.105 | 0.00407 | 758 | -0.113 | -0.097 |
|  | M08 | -0.1029 | 0.00407 | 758 | -0.1109 | -0.0949 |
|  | M09 | -0.1148 | 0.00407 | 758 | -0.1228 | -0.1068 |

**Table S15** Summary of the post-hoc Tukey test comparisons of the linear mixed-effect model used to evaluate the differences in eDNA or eRNA decay constants between gene regions (Gene) across each mussel abundance (Abundance).

| Abundance | contrast | estimate | SE | df | t.ratio | p.value |
| --- | --- | --- | --- | --- | --- | --- |
| 2 | M1-M2 | 0.003739 | 0.00424 | 758 | 0.882 | 0.9939 |
|  | M1-M3 | -0.002494 | 0.00424 | 758 | -0.588 | 0.9997 |
|  | M1-M4 | 0.008407 | 0.00427 | 758 | 1.967 | 0.5667 |
|  | M1-M5 | 0.008106 | 0.00424 | 758 | 1.912 | 0.6056 |
|  | M1-M6 | 0.009194 | 0.00427 | 758 | 2.152 | 0.4389 |
|  | M1-M7 | 0.012475 | 0.00424 | 758 | 2.943 | 0.0805 |
|  | M1-M8 | 0.019935 | 0.00424 | 758 | 4.702 | 0.0001 |
|  | M1-M9 | 0.033978 | 0.00424 | 758 | 8.015 | <.0001 |
|  | M2-M3 | -0.006233 | 0.00424 | 758 | -1.469 | 0.8696 |
|  | M2-M4 | 0.004668 | 0.00427 | 758 | 1.092 | 0.9753 |
|  | M2-M5 | 0.004367 | 0.00424 | 758 | 1.03 | 0.9829 |
|  | M2-M6 | 0.005455 | 0.00427 | 758 | 1.277 | 0.938 |
|  | M2-M7 | 0.008736 | 0.00424 | 758 | 2.061 | 0.5012 |
|  | M2-M8 | 0.016196 | 0.00424 | 758 | 3.82 | 0.0045 |
|  | M2-M9 | 0.030239 | 0.00424 | 758 | 7.133 | <.0001 |
|  | M3-M4 | 0.010901 | 0.00428 | 758 | 2.549 | 0.2104 |
|  | M3-M5 | 0.0106 | 0.00424 | 758 | 2.498 | 0.2344 |
|  | M3-M6 | 0.011688 | 0.00428 | 758 | 2.733 | 0.138 |
|  | M3-M7 | 0.014969 | 0.00424 | 758 | 3.528 | 0.0131 |
|  | M3-M8 | 0.022429 | 0.00424 | 758 | 5.286 | <.0001 |
|  | M3-M9 | 0.036472 | 0.00424 | 758 | 8.596 | <.0001 |
|  | M4-M5 | -0.000302 | 0.00427 | 758 | -0.071 | 1 |
|  | M4-M6 | 0.000787 | 0.0043 | 758 | 0.183 | 1 |
|  | M4-M7 | 0.004068 | 0.00427 | 758 | 0.952 | 0.9898 |
|  | M4-M8 | 0.011527 | 0.00427 | 758 | 2.698 | 0.1503 |
|  | M4-M9 | 0.025571 | 0.00427 | 758 | 5.984 | <.0001 |
|  | M5-M6 | 0.001088 | 0.00427 | 758 | 0.255 | 1 |
|  | M5-M7 | 0.004369 | 0.00424 | 758 | 1.031 | 0.9829 |
|  | M5-M8 | 0.011829 | 0.00424 | 758 | 2.79 | 0.1199 |
|  | M5-M9 | 0.025872 | 0.00424 | 758 | 6.103 | <.0001 |
|  | M6-M7 | 0.003281 | 0.00427 | 758 | 0.768 | 0.9977 |
|  | M6-M8 | 0.010741 | 0.00427 | 758 | 2.514 | 0.227 |
|  | M6-M9 | 0.024784 | 0.00427 | 758 | 5.8 | <.0001 |
|  | M7-M8 | 0.007459 | 0.00424 | 758 | 1.76 | 0.7089 |
|  | M7-M9 | 0.021503 | 0.00424 | 758 | 5.072 | <.0001 |
|  | M8-M9 | 0.014043 | 0.00424 | 758 | 3.313 | 0.0269 |
| 6 | M1-M2 | 0.003854 | 0.00394 | 758 | 0.978 | 0.9878 |
|  | M1-M3 | -0.001361 | 0.00394 | 758 | -0.345 | 1 |
|  | M1-M4 | 0.008502 | 0.00395 | 758 | 2.154 | 0.4372 |
|  | M1-M5 | 0.00816 | 0.00394 | 758 | 2.071 | 0.4941 |
|  | M1-M6 | 0.009455 | 0.00395 | 758 | 2.395 | 0.2878 |
|  | M1-M7 | 0.013926 | 0.00394 | 758 | 3.534 | 0.0128 |
|  | M1-M8 | 0.020556 | 0.00394 | 758 | 5.217 | <.0001 |
|  | M1-M9 | 0.034415 | 0.00394 | 758 | 8.735 | <.0001 |
|  | M2-M3 | -0.005215 | 0.00394 | 758 | -1.324 | 0.9242 |
|  | M2-M4 | 0.004648 | 0.00395 | 758 | 1.178 | 0.9611 |
|  | M2-M5 | 0.004305 | 0.00394 | 758 | 1.093 | 0.9753 |
|  | M2-M6 | 0.005601 | 0.00395 | 758 | 1.419 | 0.8905 |
|  | M2-M7 | 0.010072 | 0.00394 | 758 | 2.556 | 0.2072 |
|  | M2-M8 | 0.016701 | 0.00394 | 758 | 4.239 | 0.0008 |
|  | M2-M9 | 0.03056 | 0.00394 | 758 | 7.756 | <.0001 |
|  | M3-M4 | 0.009863 | 0.00395 | 758 | 2.499 | 0.2341 |
|  | M3-M5 | 0.00952 | 0.00394 | 758 | 2.416 | 0.2764 |
|  | M3-M6 | 0.010816 | 0.00395 | 758 | 2.74 | 0.1357 |
|  | M3-M7 | 0.015287 | 0.00394 | 758 | 3.88 | 0.0036 |
|  | M3-M8 | 0.021916 | 0.00394 | 758 | 5.562 | <.0001 |
|  | M3-M9 | 0.035775 | 0.00394 | 758 | 9.08 | <.0001 |
|  | M4-M5 | -0.000343 | 0.00395 | 758 | -0.087 | 1 |
|  | M4-M6 | 0.000953 | 0.00395 | 758 | 0.241 | 1 |
|  | M4-M7 | 0.005424 | 0.00395 | 758 | 1.374 | 0.9074 |
|  | M4-M8 | 0.012053 | 0.00395 | 758 | 3.054 | 0.059 |
|  | M4-M9 | 0.025912 | 0.00395 | 758 | 6.565 | <.0001 |
|  | M5-M6 | 0.001295 | 0.00395 | 758 | 0.328 | 1 |
|  | M5-M7 | 0.005766 | 0.00394 | 758 | 1.463 | 0.872 |
|  | M5-M8 | 0.012396 | 0.00394 | 758 | 3.146 | 0.045 |
|  | M5-M9 | 0.026255 | 0.00394 | 758 | 6.664 | <.0001 |
|  | M6-M7 | 0.004471 | 0.00395 | 758 | 1.133 | 0.9692 |
|  | M6-M8 | 0.011101 | 0.00395 | 758 | 2.812 | 0.1134 |
|  | M6-M9 | 0.02496 | 0.00395 | 758 | 6.324 | <.0001 |
|  | M7-M8 | 0.00663 | 0.00394 | 758 | 1.683 | 0.7572 |
|  | M7-M9 | 0.020489 | 0.00394 | 758 | 5.2 | <.0001 |
|  | M8-M9 | 0.013859 | 0.00394 | 758 | 3.517 | 0.0136 |
| 12 | M1-M2 | 0.004028 | 0.00362 | 758 | 1.112 | 0.9725 |
|  | M1-M3 | 0.000339 | 0.00363 | 758 | 0.093 | 1 |
|  | M1-M4 | 0.008645 | 0.00366 | 758 | 2.365 | 0.3049 |
|  | M1-M5 | 0.008241 | 0.00362 | 758 | 2.274 | 0.3591 |
|  | M1-M6 | 0.009847 | 0.00366 | 758 | 2.694 | 0.1516 |
|  | M1-M7 | 0.016102 | 0.00362 | 758 | 4.444 | 0.0003 |
|  | M1-M8 | 0.021487 | 0.00362 | 758 | 5.93 | <.0001 |
|  | M1-M9 | 0.03507 | 0.00362 | 758 | 9.679 | <.0001 |
|  | M2-M3 | -0.003688 | 0.00363 | 758 | -1.016 | 0.9844 |
|  | M2-M4 | 0.004617 | 0.00366 | 758 | 1.263 | 0.9415 |
|  | M2-M5 | 0.004213 | 0.00362 | 758 | 1.163 | 0.9639 |
|  | M2-M6 | 0.005819 | 0.00366 | 758 | 1.592 | 0.8092 |
|  | M2-M7 | 0.012075 | 0.00362 | 758 | 3.333 | 0.0252 |
|  | M2-M8 | 0.017459 | 0.00362 | 758 | 4.819 | 0.0001 |
|  | M2-M9 | 0.031042 | 0.00362 | 758 | 8.567 | <.0001 |
|  | M3-M4 | 0.008306 | 0.00366 | 758 | 2.268 | 0.3633 |
|  | M3-M5 | 0.007902 | 0.00363 | 758 | 2.176 | 0.4226 |
|  | M3-M6 | 0.009508 | 0.00366 | 758 | 2.596 | 0.19 |
|  | M3-M7 | 0.015763 | 0.00363 | 758 | 4.341 | 0.0005 |
|  | M3-M8 | 0.021148 | 0.00363 | 758 | 5.823 | <.0001 |
|  | M3-M9 | 0.034731 | 0.00363 | 758 | 9.564 | <.0001 |
|  | M4-M5 | -0.000404 | 0.00366 | 758 | -0.111 | 1 |
|  | M4-M6 | 0.001202 | 0.00368 | 758 | 0.326 | 1 |
|  | M4-M7 | 0.007457 | 0.00366 | 758 | 2.04 | 0.5155 |
|  | M4-M8 | 0.012842 | 0.00366 | 758 | 3.514 | 0.0138 |
|  | M4-M9 | 0.026425 | 0.00366 | 758 | 7.23 | <.0001 |
|  | M5-M6 | 0.001606 | 0.00366 | 758 | 0.439 | 1 |
|  | M5-M7 | 0.007861 | 0.00362 | 758 | 2.17 | 0.4267 |
|  | M5-M8 | 0.013246 | 0.00362 | 758 | 3.656 | 0.0083 |
|  | M5-M9 | 0.026829 | 0.00362 | 758 | 7.405 | <.0001 |
|  | M6-M7 | 0.006255 | 0.00366 | 758 | 1.711 | 0.7395 |
|  | M6-M8 | 0.01164 | 0.00366 | 758 | 3.185 | 0.0401 |
|  | M6-M9 | 0.025223 | 0.00366 | 758 | 6.901 | <.0001 |
|  | M7-M8 | 0.005385 | 0.00362 | 758 | 1.486 | 0.862 |
|  | M7-M9 | 0.018968 | 0.00362 | 758 | 5.235 | <.0001 |
|  | M8-M9 | 0.013583 | 0.00362 | 758 | 3.749 | 0.0059 |
| 24 | M1-M2 | 0.004374 | 0.0036 | 758 | 1.217 | 0.9528 |
|  | M1-M3 | 0.003739 | 0.00367 | 758 | 1.018 | 0.9841 |
|  | M1-M4 | 0.00893 | 0.00392 | 758 | 2.275 | 0.3585 |
|  | M1-M5 | 0.008403 | 0.0036 | 758 | 2.337 | 0.321 |
|  | M1-M6 | 0.01063 | 0.00392 | 758 | 2.709 | 0.1464 |
|  | M1-M7 | 0.020455 | 0.0036 | 758 | 5.69 | <.0001 |
|  | M1-M8 | 0.02335 | 0.0036 | 758 | 6.495 | <.0001 |
|  | M1-M9 | 0.03638 | 0.0036 | 758 | 10.12 | <.0001 |
|  | M2-M3 | -0.000635 | 0.00367 | 758 | -0.173 | 1 |
|  | M2-M4 | 0.004555 | 0.00392 | 758 | 1.161 | 0.9643 |
|  | M2-M5 | 0.004029 | 0.0036 | 758 | 1.121 | 0.9711 |
|  | M2-M6 | 0.006256 | 0.00392 | 758 | 1.594 | 0.8081 |
|  | M2-M7 | 0.01608 | 0.0036 | 758 | 4.473 | 0.0003 |
|  | M2-M8 | 0.018976 | 0.0036 | 758 | 5.278 | <.0001 |
|  | M2-M9 | 0.032006 | 0.0036 | 758 | 8.903 | <.0001 |
|  | M3-M4 | 0.00519 | 0.00399 | 758 | 1.301 | 0.9312 |
|  | M3-M5 | 0.004664 | 0.00367 | 758 | 1.27 | 0.9398 |
|  | M3-M6 | 0.006891 | 0.00399 | 758 | 1.727 | 0.7299 |
|  | M3-M7 | 0.016716 | 0.00367 | 758 | 4.552 | 0.0002 |
|  | M3-M8 | 0.019611 | 0.00367 | 758 | 5.34 | <.0001 |
|  | M3-M9 | 0.032641 | 0.00367 | 758 | 8.888 | <.0001 |
|  | M4-M5 | -0.000527 | 0.00392 | 758 | -0.134 | 1 |
|  | M4-M6 | 0.001701 | 0.00421 | 758 | 0.404 | 1 |
|  | M4-M7 | 0.011525 | 0.00392 | 758 | 2.937 | 0.0818 |
|  | M4-M8 | 0.01442 | 0.00392 | 758 | 3.674 | 0.0078 |
|  | M4-M9 | 0.02745 | 0.00392 | 758 | 6.994 | <.0001 |
|  | M5-M6 | 0.002228 | 0.00392 | 758 | 0.568 | 0.9997 |
|  | M5-M7 | 0.012052 | 0.0036 | 758 | 3.352 | 0.0237 |
|  | M5-M8 | 0.014947 | 0.0036 | 758 | 4.158 | 0.0012 |
|  | M5-M9 | 0.027977 | 0.0036 | 758 | 7.782 | <.0001 |
|  | M6-M7 | 0.009824 | 0.00392 | 758 | 2.503 | 0.2319 |
|  | M6-M8 | 0.01272 | 0.00392 | 758 | 3.241 | 0.0337 |
|  | M6-M9 | 0.025749 | 0.00392 | 758 | 6.561 | <.0001 |
|  | M7-M8 | 0.002895 | 0.0036 | 758 | 0.805 | 0.9967 |
|  | M7-M9 | 0.015925 | 0.0036 | 758 | 4.43 | 0.0004 |
|  | M8-M9 | 0.01303 | 0.0036 | 758 | 3.624 | 0.0093 |
| 48 | M1-M2 | 0.005067 | 0.00556 | 758 | 0.912 | 0.9923 |
|  | M1-M3 | 0.010539 | 0.00583 | 758 | 1.808 | 0.6767 |
|  | M1-M4 | 0.0095 | 0.00674 | 759 | 1.41 | 0.8942 |
|  | M1-M5 | 0.008727 | 0.00556 | 758 | 1.571 | 0.8206 |
|  | M1-M6 | 0.012198 | 0.00674 | 759 | 1.81 | 0.6757 |
|  | M1-M7 | 0.02916 | 0.00556 | 758 | 5.248 | <.0001 |
|  | M1-M8 | 0.027076 | 0.00556 | 758 | 4.873 | <.0001 |
|  | M1-M9 | 0.039 | 0.00556 | 758 | 7.019 | <.0001 |
|  | M2-M3 | 0.005472 | 0.00583 | 758 | 0.939 | 0.9907 |
|  | M2-M4 | 0.004432 | 0.00674 | 759 | 0.658 | 0.9992 |
|  | M2-M5 | 0.00366 | 0.00556 | 758 | 0.659 | 0.9992 |
|  | M2-M6 | 0.00713 | 0.00674 | 759 | 1.058 | 0.9798 |
|  | M2-M7 | 0.024092 | 0.00556 | 758 | 4.336 | 0.0006 |
|  | M2-M8 | 0.022009 | 0.00556 | 758 | 3.961 | 0.0026 |
|  | M2-M9 | 0.033933 | 0.00556 | 758 | 6.107 | <.0001 |
|  | M3-M4 | -0.00104 | 0.00695 | 759 | -0.15 | 1 |
|  | M3-M5 | -0.001812 | 0.00583 | 758 | -0.311 | 1 |
|  | M3-M6 | 0.001658 | 0.00695 | 759 | 0.239 | 1 |
|  | M3-M7 | 0.018621 | 0.00583 | 758 | 3.195 | 0.0389 |
|  | M3-M8 | 0.016537 | 0.00583 | 758 | 2.837 | 0.1063 |
|  | M3-M9 | 0.028461 | 0.00583 | 758 | 4.883 | <.0001 |
|  | M4-M5 | -0.000773 | 0.00674 | 759 | -0.115 | 1 |
|  | M4-M6 | 0.002698 | 0.00768 | 758 | 0.351 | 1 |
|  | M4-M7 | 0.01966 | 0.00674 | 759 | 2.917 | 0.0862 |
|  | M4-M8 | 0.017577 | 0.00674 | 759 | 2.608 | 0.1849 |
|  | M4-M9 | 0.0295 | 0.00674 | 759 | 4.377 | 0.0005 |
|  | M5-M6 | 0.003471 | 0.00674 | 759 | 0.515 | 0.9999 |
|  | M5-M7 | 0.020433 | 0.00556 | 758 | 3.678 | 0.0077 |
|  | M5-M8 | 0.018349 | 0.00556 | 758 | 3.303 | 0.0278 |
|  | M5-M9 | 0.030273 | 0.00556 | 758 | 5.449 | <.0001 |
|  | M6-M7 | 0.016962 | 0.00674 | 759 | 2.517 | 0.2254 |
|  | M6-M8 | 0.014879 | 0.00674 | 759 | 2.208 | 0.4016 |
|  | M6-M9 | 0.026802 | 0.00674 | 759 | 3.977 | 0.0025 |
|  | M7-M8 | -0.002084 | 0.00556 | 758 | -0.375 | 1 |
|  | M7-M9 | 0.00984 | 0.00556 | 758 | 1.771 | 0.7014 |
|  | M8-M9 | 0.011924 | 0.00556 | 758 | 2.146 | 0.4426 |


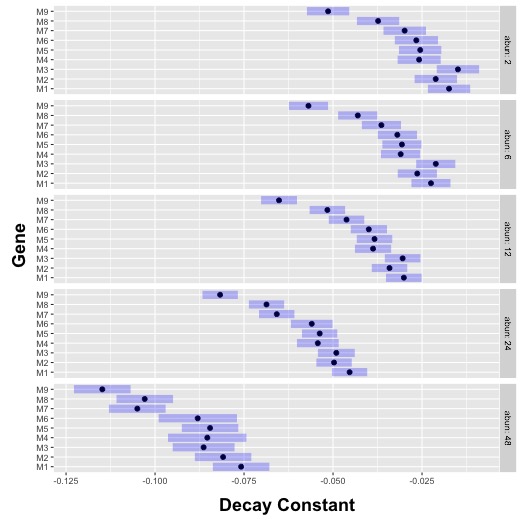


**Figure S3** Relationship between mussel abundance and eDNA or eRNA decay constant for each of the target gene regions estimated from the linear mixed-effects model.

Next, the absolute estimates for the eDNA or eRNA decay constants for each mussel abundance and gene fragment was obtained by fitting a linear model for each abundance treatment by gene region combination. The linear model used the values of log_e_(C*_t_*/C_0_) as the response variable (y) across the first 72 hours as given in equation S4. The model summary is given in Table S16 and shows the data used to construct Figure 2.

(S4) model<-lm(log(y)~0+Time, data=data)

**Table S16** Summary of the linear models used to estimate the eDNA or eRNA decay constant for each mussel abundance treatment by gene region.

| Abundance | Gene Region | Estimate | SE | t value | Pr(>\|t\|) | R-squared |
| --- | --- | --- | --- | --- | --- | --- |
| D1 | M1 | -0.018 | 0.003 | -5.765 | 2.29E-05 | 0.642 |
|  | M2 | -0.017 | 0.003 | -6.033 | 1.34E-05 | 0.663 |
|  | M3 | -0.008 | 0.004 | -1.898 | 7.41E-02 | 0.126 |
|  | M4 | -0.021 | 0.002 | -9.169 | 5.44E-08 | 0.822 |
|  | M5 | -0.015 | 0.002 | -7.998 | 3.66E-07 | 0.778 |
|  | M6 | -0.022 | 0.002 | -9.169 | 5.44E-08 | 0.822 |
|  | M7 | -0.025 | 0.003 | -8.245 | 2.41E-07 | 0.788 |
|  | M8 | -0.030 | 0.003 | -8.908 | 8.20E-08 | 0.813 |
|  | M9 | -0.046 | 0.003 | -14.245 | 7.01E-11 | 0.918 |
| D2 | M1 | -0.027 | 0.004 | -6.314 | 5.72E-05 | 0.764 |
|  | M2 | -0.031 | 0.006 | -5.383 | 2.23E-04 | 0.700 |
|  | M3 | -0.022 | 0.004 | -5.239 | 2.77E-04 | 0.688 |
|  | M4 | -0.039 | 0.004 | -10.554 | 4.30E-07 | 0.902 |
|  | M5 | -0.041 | 0.004 | -11.056 | 2.69E-07 | 0.910 |
|  | M6 | -0.040 | 0.004 | -10.554 | 4.30E-07 | 0.902 |
|  | M7 | -0.038 | 0.004 | -9.067 | 1.95E-06 | 0.871 |
|  | M8 | -0.047 | 0.005 | -8.741 | 2.79E-06 | 0.863 |
|  | M9 | -0.063 | 0.005 | -12.654 | 6.73E-08 | 0.930 |
| D3 | M1 | -0.030 | 0.003 | -11.327 | 2.42E-09 | 0.876 |
|  | M2 | -0.028 | 0.004 | -6.615 | 4.38E-06 | 0.704 |
|  | M3 | -0.044 | 0.004 | -11.008 | 3.72E-09 | 0.870 |
|  | M4 | -0.046 | 0.003 | -16.687 | 5.64E-12 | 0.939 |
|  | M5 | -0.046 | 0.003 | -15.099 | 2.79E-11 | 0.927 |
|  | M6 | -0.048 | 0.003 | -16.687 | 5.64E-12 | 0.939 |
|  | M7 | -0.042 | 0.002 | -21.018 | 1.33E-13 | 0.961 |
|  | M8 | -0.050 | 0.002 | -21.761 | 7.50E-14 | 0.963 |
|  | M9 | -0.072 | 0.004 | -19.226 | 5.70E-13 | 0.953 |
| D4 | M1 | -0.052 | 0.004 | -12.714 | 6.41E-08 | 0.930 |
|  | M2 | -0.064 | 0.007 | -8.653 | 3.07E-06 | 0.860 |
|  | M3 | -0.050 | 0.007 | -7.607 | 1.83E-05 | 0.838 |
|  | M4 | -0.066 | 0.010 | -6.869 | 4.35E-05 | 0.808 |
|  | M5 | -0.067 | 0.010 | -6.486 | 4.52E-05 | 0.774 |
|  | M6 | -0.068 | 0.010 | -6.869 | 4.35E-05 | 0.808 |
|  | M7 | -0.072 | 0.005 | -14.158 | 2.09E-08 | 0.943 |
|  | M8 | -0.074 | 0.002 | -33.020 | 2.35E-12 | 0.989 |
|  | M9 | -0.101 | 0.005 | -22.206 | 1.73E-10 | 0.976 |
| D5 | M1 | -0.081 | 0.006 | -12.701 | 4.20E-10 | 0.899 |
|  | M2 | -0.081 | 0.005 | -15.859 | 1.28E-11 | 0.933 |
|  | M3 | -0.089 | 0.013 | -6.743 | 2.07E-05 | 0.774 |
|  | M4 | -0.077 | 0.010 | -7.832 | 2.82E-06 | 0.812 |
|  | M5 | -0.083 | 0.005 | -15.683 | 1.52E-11 | 0.932 |
|  | M6 | -0.079 | 0.010 | -7.832 | 2.82E-06 | 0.812 |
|  | M7 | -0.104 | 0.007 | -14.867 | 3.57E-11 | 0.924 |
|  | M8 | -0.097 | 0.006 | -16.047 | 5.99E-10 | 0.948 |
|  | M9 | -0.115 | 0.006 | -18.897 | 7.55E-13 | 0.952 |

**Figure S4** The best fit line of the first-order exponential decay model (regression line over the first 72 hours) for all eDNA and eRNA fragments plotted across the 240 hour sampling time. The eDNA or eRNA fragments given as the genetic target region (i.e. 16S, COI, 18S, or H2B) with the fragment size as the total number of base pairs (bp).
